# Supplementary material for: Bifidobacterium lactis Probio-M8 Adjuvant Treatment Confers Added Benefits to Patients with Coronary Artery Disease via Target Modulation of the Gut-Heart/-Brain Axes
Source: mSystems. 2022 Mar 28;7(2):e00100-22. doi: 10.1128/msystems.00100-22 (PMC9040731; doi:10.1128/msystems.00100-22)
Supplement: TABLE S3 [file msystems.00100-22-st003.pdf]

Table S3. Clinical indicators for coronary artery disease measured before and after the trial (n=60)

	Probiotic group					Placebo group					Probiotic vs Placebo	
	**Result statistics**				**Corrected P-value, T-test**	**Result statistics**				**Corrected P-value, T-test**	**0d Corrected P-value, Wilcoxon-test**	**180d Corrected P-value, Wilcoxon-test**
Differential indicators	Mean_pro_0d	Mean_pro_180d	SD_pro_0d	SD_pro_180d	pro_0d vs pro_180d	Mean_pla_0d	Mean_pla_180d	SD_pla_0d	SD_pla_180d	pla_0d vs pla_180d	0d	180d
Anginal Frequency	49.89	71.00	7.46	7.04	<2E-16	46.84	62.83	5.41	5.45	5.50E-11	0.10	3.00E-05
Anginal Stability	43.13	62.25	4.90	6.66	<2E-16	42.53	55.07	5.38	5.23	2.00E-12	0.66	1.90E-04
Disease Perception	43.03	65.60	4.04	4.53	<2E-16	43.44	66.87	5.22	5.00	7.00E-13	0.82	1.90E-01
Physical Limitation	43.60	67.18	4.38	5.79	<2E-16	45.86	57.59	5.89	5.98	3.90E-12	0.15	9.70E-07
Treatment Satisfaction	44.03	69.47	4.53	4.95	<2E-16	43.78	64.32	4.31	5.02	2.90E-13	0.93	9.00E-04
Self-rating Anxiety Scale	46.38	38.38	4.86	4.06	2.00E-11	48.16	42.10	4.09	3.51	1.50E-09	0.15	1.90E-04
Self-rating Depression Scale	50.28	39.27	5.16	4.28	2.80E-16	50.54	46.72	3.66	4.02	0.0015	0.49	2.30E-07
Interleukin-6 (pg/mL)	46.19	30.77	5.08	4.64	<2E-16	45.97	34.59	4.47	3.22	3.30E-11	0.89	1.50E-03

Low-density lipoprotein cholesterol (mmol/L)	3.41	2.13	0.38	0.19	<2E-16	3.57	2.62	0.24	0.19	4.30E-16	0.23	1.60E-10
Cereal third transaminase (u/L)	21.92	22.79	14.73	12.26	1	23.98	23.42	15.22	11.16	1	1	1
Blood urea nitrogen (mmol/L)	5.28	5.13	1.27	0.94	1	5.37	6.85	1.46	8.04	1	1	1
Creatinine (ummol/L)	60.49	60.58	12.93	10.59	1	68.89	68.31	21.49	18.44	0.78	0.78	0.78
White blood cell count (*10^9/L)	5.91	5.93	1.47	1.16	1	5.91	6.23	1.41	0.92	0.49	1	0.49
