# Supplementary material for: Bifidobacterium lactis Probio-M8 Adjuvant Treatment Confers Added Benefits to Patients with Coronary Artery Disease via Target Modulation of the Gut-Heart/-Brain Axes
Source: mSystems. 2022 Mar 28;7(2):e00100-22. doi: 10.1128/msystems.00100-22 (PMC9040731; doi:10.1128/msystems.00100-22)
Supplement: TABLE S4 [file msystems.00100-22-st004.pdf]

Sample_A02_2.bin_1	A022.M001	40	4.69	171.34	53.85	249.75	99.28	0.30	Enterobacteriaceae	Proteobacteria	Citrobacter koseri
Sample_A19_1.bin_1	A191.M001	36	4.26	164.43	46.33	234.87	98.51	0.00	Bacteroidales	Bacteroidetes	Bacteroides uniformis
Sample_A32_1.bin_1	A321.M001	29	2.66	159.61	41.62	216.80	98.63	0.00	Lachnospiraceae	Firmicutes	Eubacterium rectale
Sample_A24_1.bin_1	A241.M001	96	2.87	39.84	50.62	211.49	98.42	0.12	Bacteroidales	Bacteroidetes	Prevotella sp. CAG:520
Sample_A24_2.bin_1	A242.M001	43	1.99	61.96	59.96	199.93	93.95	1.17	Clostridiales	Firmicutes	Clostridiales bacterium
Sample_B22_2.bin_1	B222.M001	38	5.47	236.78	57.43	175.54	98.80	0.92	Enterobacteriaceae	Proteobacteria	Klebsiella pneumoniae
Sample_B20_2.bin_1	B202.M001	15	1.16	126.82	26.47	173.54	91.57	2.25	Bacteria	Firmicutes	Firmicutes bacterium CAG:460
Sample_A17_3.bin_1	A173.M001	44	3.61	124.46	44.42	170.00	96.34	4.00	Bacteroidales	Bacteroidetes	Bacteroides eggerthii
Sample_A22_1.bin_1	A221.M001	82	3.01	56.08	49.57	164.98	96.98	0.49	Bacteroidales	Bacteroidetes	Prevotella sp. 885
Sample_A09_2.bin_1	A092.M001	52	2.54	58.74	54.35	158.79	97.09	0.00	Prevotella	Bacteroidetes	Prevotella sp.
Sample_B16_1.bin_1	B161.M001	114	2.46	36.62	44.79	154.07	93.09	0.00	Bacteroidales	Bacteroidetes	Prevotella sp. CAG:5226
Sample_A31_1.bin_1	A311.M001	16	2.81	262.18	43.20	152.76	97.65	0.00	Clostridiales	Firmicutes	Coprococcus eutactus CAG:665
Sample_A07_3.bin_1	A073.M001	31	3.05	125.40	44.53	151.70	97.46	4.11	Clostridiales	Firmicutes	uncultured Blautia sp.
Sample_B16_3.bin_1	B163.M001	39	3.28	110.47	46.07	149.11	91.09	0.00	Bacteroidales	Bacteroidetes	Bacteroides stercoris
Sample_A07_1.bin_1	A071.M001	12	1.75	240.43	31.08	138.54	100.00	0.00	Euryarchaeota	Euryarchaeota	Methanobrevibacter smithii CAG:186

Sample_A05_3.bin_1	A053.M001	99	3.06	40.77	40.80	135.99	93.11	0.37	Bacteroidales	Bacteroidetes	Bacteroides coprocola CAG:162
Sample_A06_3.bin_1	A063.M001	42	5.20	238.16	57.97	134.73	98.71	0.46	Enterobacteriaceae	Proteobacteria	Klebsiella pneumoniae
Sample_B14_2.bin_1	B142.M001	21	1.87	220.85	43.82	134.00	97.31	0.00	Clostridiales	Firmicutes	Anaerotruncus sp. CAG:528
Sample_A33_1.bin_1	A331.M001	103	3.16	43.50	43.19	133.89	96.41	0.63	Clostridiales	Firmicutes	Clostridium sp. TM06-18
Sample_B02_2.bin_1	B022.M001	33	2.07	84.45	39.14	132.97	98.65	0.34	Clostridiales	Firmicutes	Ruminococcaceae bacterium
Sample_A30_2.bin_1	A302.M001	57	2.71	80.73	46.78	132.70	97.98	0.67	Clostridiales	Firmicutes	uncultured Ruminococcus sp.
Sample_A17_2.bin_1	A172.M001	79	3.36	72.91	51.19	131.62	98.64	0.34	Prevotella	Bacteroidetes	Prevotella sp.
Sample_B23_3.bin_1	B233.M001	27	2.74	137.81	46.17	131.34	97.40	2.03	Lachnospiraceae	Firmicutes	Clostridium sp. CAG:510
Sample_A32_2.bin_1	A322.M001	73	3.02	53.56	44.58	126.30	94.77	0.00	Bacteroidales	Bacteroidetes	Bacteroides plebeius
Sample_B21_1.bin_1	B211.M001	20	2.68	230.00	42.96	126.21	91.94	2.68	Clostridiales	Firmicutes	Coprococcus eutactus
Sample_A02_3.bin_1	A023.M001	47	2.74	95.76	35.26	122.87	90.26	3.58	Clostridiales	Firmicutes	Lachnospiraceae bacterium
Sample_A29_1.bin_1	A291.M001	27	2.01	121.87	41.80	114.00	98.69	0.26	Lactobacillales	Firmicutes	Lactobacillus agilis
Sample_A18_2.bin_2	A182.M002	86	2.82	52.64	48.27	112.61	96.66	0.12	Bacteroidales	Bacteroidetes	Prevotella sp. CAG:592
Sample_B11_1.bin_2	B111.M002	29	2.58	151.27	48.57	112.43	99.36	0.00	Clostridiales	Firmicutes	Clostridium sp. AM28-20LB
Sample_B18_2.bin_2	B182.M002	10	1.20	402.08	27.58	108.71	92.41	1.12	Bacteria	Firmicutes	Clostridium sp. CAG:594

Sample_A06_3.bin_2	A063.M002	28	1.40	128.67	28.02	105.66	80.16	2.45	Clostridiales	Firmicutes	Clostridiales bacterium
Sample_B02_1.bin_2	B021.M002	58	2.42	56.75	58.41	103.60	91.27	1.34	Clostridiales	Firmicutes	Firmicutes bacterium CAG:124
Sample_A09_3.bin_2	A093.M002	89	2.15	39.87	52.11	101.39	100.00	0.63	Firmicutes	Firmicutes	Dialister sp. CAG:486
Sample_A30_1.bin_2	A301.M002	30	5.15	297.02	57.64	101.33	100.00	0.19	Enterobacteriaceae	Proteobacteria	Klebsiella pneumoniae
Sample_A33_1.bin_2	A331.M002	22	2.29	196.68	54.22	100.81	99.51	0.96	Bacteroidetes	Bacteroidetes	Alistipes putredinis
Sample_A12_3.bin_2	A123.M002	31	2.17	122.21	39.87	97.75	98.52	3.47	Streptococcus	Firmicutes	Streptococcus salivarius
Sample_A06_1.bin_2	A061.M002	21	1.88	162.65	32.88	95.79	88.88	0.85	Bacteria	Candidatus Melainabacteria	Candidatus Gastranaerophilales bacterium HUM_2
Sample_A04_2.bin_2	A042.M002	107	2.76	41.13	41.90	95.77	96.86	0.00	Clostridiales	Firmicutes	uncultured Ruminococcus sp.
Sample_A32_3.bin_2	A323.M002	50	1.28	47.04	25.74	94.76	93.25	2.81	Bacteria	Firmicutes	Clostridium sp. CAG:914
Sample_B23_3.bin_2	B233.M002	23	3.72	231.38	48.43	92.21	98.51	0.00	Bacteroidales	Bacteroidetes	Paraprevotella clara CAG:116
Sample_B13_2.bin_2	B132.M002	25	2.83	177.51	47.12	85.99	93.67	0.00	Clostridiales	Firmicutes	Clostridium sp. TM06-18
Sample_A19_1.bin_2	A191.M002	8	1.53	211.71	47.36	85.89	92.40	0.70	Clostridiales	Firmicutes	Clostridiales bacterium
Sample_A05_2.bin_2	A052.M002	62	2.34	56.86	57.11	85.86	89.70	0.00	Clostridiales	Firmicutes	Firmicutes bacterium CAG:110
Sample_A17_3.bin_2	A173.M002	66	2.44	59.77	30.10	85.77	95.28	0.00	Bacteria	Firmicutes	Coprobacillus sp. 8_1_38FAA
Sample_A31_3.bin_2	A313.M002	50	2.25	69.94	31.52	83.45	100.00	0.63	Firmicutes	Firmicutes	Megamonas funiformis

Sample_A02_3.bin_2	A023.M002	41	2.16	86.57	59.88	82.82	98.38	1.34	Actinobacteria	Actinobacteria	Collinsella aerofaciens
Sample_B03_2.bin_2	B032.M002	71	3.00	57.58	43.30	82.19	91.31	1.29	Clostridiales	Firmicutes	uncultured Blautia sp.
Sample_B20_3.bin_2	B203.M002	22	2.41	163.01	44.43	81.73	100.00	0.00	Clostridiales	Firmicutes	Clostridium sp. CAG:632
Sample_B12_2.bin_2	B122.M002	40	2.32	81.19	40.89	80.50	96.00	0.00	Lachnospiraceae	Firmicutes	Ruminococcus torques
Sample_A29_3.bin_2	A293.M002	82	3.42	68.52	59.26	80.47	98.65	1.51	Clostridiales	Firmicutes	Ruminococcaceae bacterium
Sample_A07_2.bin_2	A072.M002	57	2.99	72.45	44.82	79.54	97.83	0.95	Clostridiales	Firmicutes	Blautia sp. OM07-19
Sample_A27_2.bin_2	A272.M002	17	1.75	157.00	49.91	79.24	94.63	0.00	Clostridiales	Firmicutes	Ruminococcaceae bacterium
Sample_B20_1.bin_2	B201.M002	16	1.27	160.62	28.20	78.91	80.02	0.22	Clostridiales	Firmicutes	Clostridium sp. CAG:452
Sample_A01_3.bin_2	A013.M002	69	3.16	65.92	47.01	78.65	97.42	1.01	Lachnospiraceae	Firmicutes	uncultured Clostridium sp.
Sample_A02_1.bin_2	A021.M002	71	2.33	50.95	38.33	78.56	98.75	0.00	Lactobacillales	Firmicutes	Enterococcus faecium
Sample_A09_1.bin_2	A091.M002	33	2.50	135.91	49.97	77.74	98.30	0.00	Bacteroidales	Bacteroidetes	Porphyromonadaceae bacterium
Sample_B22_2.bin_2	B222.M002	10	1.85	311.24	53.85	77.40	97.31	0.00	Clostridiales	Firmicutes	Clostridiales bacterium
Sample_B21_2.bin_3	B212.M003	13	2.11	453.31	56.25	76.35	100.00	0.23	Bifidobacteriaceae	Actinobacteria	Bifidobacterium pseudocatenulatum
Sample_B15_3.bin_3	B153.M003	36	5.90	224.78	55.01	75.64	98.80	0.51	Enterobacteriaceae	Proteobacteria	Klebsiella oxytoca
Sample_B20_2.bin_3	B202.M003	91	1.99	30.36	60.86	74.18	85.96	3.74	Clostridiales	Firmicutes	Faecalibacterium prausnitzii

Sample_A18_2.bin_3	A182.M003	85	1.84	26.70	48.84	73.00	92.40	0.00	Firmicutes	Firmicutes	Dialister sp. CAG:357
Sample_B14_2.bin_3	B142.M003	54	2.13	56.91	52.42	71.91	95.74	1.01	Clostridiales	Firmicutes	Clostridiales bacterium
Sample_A07_3.bin_3	A073.M003	96	2.91	42.14	49.54	70.42	96.34	0.00	Bacteroidales	Bacteroidetes	Prevotella sp. CAG:5226
Sample_A32_3.bin_3	A323.M003	42	4.75	176.42	41.43	70.36	98.12	1.21	Bacteroidales	Bacteroidetes	Bacteroides dorei
Sample_A09_2.bin_3	A092.M003	29	2.13	110.08	44.69	70.17	98.46	0.61	Lactobacillales	Firmicutes	Weissella confusa
Sample_A24_3.bin_3	A243.M003	21	3.12	340.20	58.78	70.09	99.27	0.48	Bacteroidetes	Bacteroidetes	Alistipes onderdonkii
Sample_B06_1.bin_3	B061.M003	53	2.16	61.29	60.07	68.45	100.00	0.81	Actinobacteria	Actinobacteria	Collinsella aerofaciens
Sample_B21_1.bin_3	B211.M003	39	4.72	145.91	54.74	67.69	96.79	4.97	Enterobacteriaceae	Proteobacteria	Enterobacter sp. Z1
Sample_A23_3.bin_3	A233.M003	107	2.42	37.22	50.83	67.07	96.64	0.00	Clostridiales	Firmicutes	Ruminococcus sp. OM06-36AC
Sample_A31_3.bin_3	A313.M003	35	2.46	122.02	58.23	66.51	97.95	0.00	Clostridiales	Firmicutes	Faecalibacterium prausnitzii
Sample_A17_1.bin_3	A171.M003	62	2.02	44.82	42.19	66.13	95.63	3.69	Streptococcus	Firmicutes	Streptococcus parasanguinis
Sample_A29_2.bin_3	A292.M003	30	1.81	83.86	57.22	65.75	88.33	1.17	Clostridiales	Firmicutes	Oscillibacter sp.
Sample_A07_1.bin_3	A071.M003	60	4.41	117.26	52.03	65.29	94.76	0.14	Enterobacteriaceae	Proteobacteria	Citrobacter freundii
Sample_A32_2.bin_3	A322.M003	29	2.03	88.28	39.51	64.83	97.98	0.34	Clostridiales	Firmicutes	Eubacterium sp. OM08-24
Sample_A29_1.bin_3	A291.M003	29	2.43	176.63	32.55	64.39	97.27	0.67	Clostridiales	Firmicutes	Eubacterium sp. CAG:156

Sample_A05_2.bin_ 3	A052.M003	13	2.14	1112.88	40.54	64.34	99.32	0.67	Clostridiale s	Firmicutes	Ruminococcus bromii
Sample_A01_3.bin_ 3	A013.M003	72	2.98	54.72	41.72	64.01	96.13	0.00	Lachnospir aceae	Firmicutes	Roseburia inulinivorans
Sample_A23_2.bin_ 3	A232.M003	27	2.24	172.91	59.95	61.83	100.00	0.81	Actinobacte ria	Actinobact eria	Collinsella aerofaciens
Sample_A04_3.bin_ 3	A043.M003	52	5.27	140.54	48.97	61.68	90.50	0.06	Clostridiale s	Firmicutes	Hungatella hathewayi
Sample_B02_1.bin_ 3	B021.M003	27	2.72	196.53	37.64	61.62	99.53	0.00	Lactobacilla les	Firmicutes	Enterococcus faecalis
Sample_A31_2.bin_ 3	A312.M003	65	2.18	50.43	59.28	61.23	93.62	0.67	Clostridiale s	Firmicutes	Oscillibacter sp.
Sample_B24_1.bin_ 3	B241.M003	42	1.91	60.11	61.58	60.56	89.26	1.13	Clostridiale s	Firmicutes	Oscillibacter sp.
Sample_A18_3.bin_ 3	A183.M003	19	2.96	226.39	58.18	59.51	97.95	0.00	Bacteria	Verrucomic robia	Akkermansia muciniphila
Sample_A18_3.bin_ 4	A183.M004	48	2.20	93.41	59.92	59.00	100.00	0.81	Actinobacte ria	Actinobact eria	Collinsella aerofaciens
Sample_A06_1.bin_ 4	A061.M004	32	1.63	63.01	60.44	58.89	86.93	0.00	Clostridiale s	Firmicutes	Firmicutes bacterium CAG:176
Sample_A21_2.bin_ 4	A212.M004	19	2.73	305.61	55.32	57.52	97.95	0.00	Bacteria	Verrucomic robia	Akkermansia muciniphila
Sample_A33_3.bin_ 4	A333.M004	103	1.86	23.42	35.24	57.40	86.63	1.39	Lactobacilla les	Firmicutes	Lactococcus lactis
Sample_A28_2.bin_ 4	A282.M004	22	2.30	157.39	52.76	54.64	97.04	0.00	Clostridiale s	Firmicutes	Clostridium sp. CAG:413
Sample_B02_2.bin_ 4	B022.M004	189	2.49	18.54	56.33	53.83	95.46	0.00	Clostridiale s	Firmicutes	Ruminococcaceae bacterium
Sample_A36_1.bin_ 4	A361.M004	14	2.29	184.58	41.92	53.69	96.64	0.00	Clostridiale s	Firmicutes	Butyrivibrio sp. CAG:318

Sample_B22_1.bin_4	B221.M004	41	2.44	78.26	41.85	52.76	93.56	0.00	Lachnospiraceae	Firmicutes	Dorea longicatena
Sample_A04_1.bin_4	A041.M004	45	1.96	77.11	56.90	52.67	85.68	0.00	Clostridiales	Firmicutes	Ruminococcaceae bacterium
Sample_B03_1.bin_4	B031.M004	18	1.28	183.01	26.74	52.51	90.16	1.12	Bacteria	Firmicutes	Firmicutes bacterium
Sample_A06_3.bin_4	A063.M004	20	1.91	165.68	60.48	51.75	100.00	0.00	Bifidobacteriaceae	Actinobacteria	Bifidobacterium animalis
Sample_B17_3.bin_4	B173.M004	15	1.95	269.77	38.01	50.52	98.65	0.00	Clostridiales	Firmicutes	Eubacterium sp. CAG:251
Sample_B21_1.bin_4	B211.M004	32	2.80	148.50	59.67	50.39	100.00	0.00	Bacteroidetes	Bacteroidetes	Alistipes shahii
Sample_B13_2.bin_4	B132.M004	40	2.57	94.29	41.98	49.14	86.71	0.12	Lachnospiraceae	Firmicutes	Roseburia sp. OF03-24
Sample_B12_1.bin_4	B121.M004	35	2.23	107.05	59.77	48.99	100.00	0.81	Actinobacteria	Actinobacteria	Collinsella aerofaciens
Sample_A23_2.bin_4	A232.M004	18	2.66	225.22	45.08	47.77	98.65	1.34	Clostridiales	Firmicutes	Clostridium sp. CAG:242
Sample_B02_3.bin_4	B023.M004	20	2.66	219.64	41.26	47.52	97.98	0.00	Clostridiales	Firmicutes	Clostridium sp. AF15-41
Sample_B16_3.bin_4	B163.M004	28	2.34	159.00	50.31	47.22	97.31	0.00	Clostridiales	Firmicutes	Ruminococcus sp. CAG:579
Sample_A28_1.bin_4	A281.M004	9	1.94	236.90	36.31	46.67	98.65	0.00	Clostridiales	Firmicutes	Firmicutes bacterium CAG:341
Sample_A18_1.bin_4	A181.M004	38	5.28	308.61	50.31	46.55	96.26	0.95	Clostridiales	Firmicutes	Hungatella hathewayi
Sample_A03_2.bin_4	A032.M004	23	1.99	116.70	49.66	46.53	93.93	1.34	Clostridiales	Firmicutes	Clostridiales bacterium
Sample_A27_2.bin_4	A272.M004	92	2.18	43.71	51.19	45.98	100.00	0.63	Firmicutes	Firmicutes	Dialister succinatiphilus

Sample_A23_3.bin_5	A233.M005	24	2.57	148.90	38.86	45.07	99.36	0.00	Clostridiales	Firmicutes	Lachnospiraceae bacterium AM25-39
Sample_A09_2.bin_5	A092.M005	54	1.81	45.44	60.74	44.70	85.27	3.74	Clostridiales	Firmicutes	Faecalibacterium prausnitzii
Sample_B11_3.bin_5	B113.M005	27	2.43	134.37	46.84	44.38	100.00	0.90	Selenomonadales	Firmicutes	Phascolarctobacterium succinatutens
Sample_B15_3.bin_5	B153.M005	9	2.24	455.73	60.03	44.36	100.00	0.12	Bifidobacteriaceae	Actinobacteria	Bifidobacterium longum
Sample_A36_1.bin_5	A361.M005	8	1.85	430.84	32.16	44.01	88.88	0.85	Bacteria	Cyanobacteria	Cyanobacteria bacterium UBA10660
Sample_A27_2.bin_5	A272.M005	36	1.76	84.32	38.77	43.04	95.62	0.00	Streptococcus	Firmicutes	Streptococcus anginosus
Sample_B12_2.bin_5	B122.M005	68	7.38	150.92	46.70	42.98	95.97	3.16	Lachnospiraceae	Firmicutes	Eisenbergiella tayi
Sample_B22_2.bin_5	B222.M005	32	2.47	114.59	48.75	42.86	100.00	0.11	Proteobacteria	Proteobacteria	Burkholderiales bacterium
Sample_A07_3.bin_5	A073.M005	44	2.70	91.52	44.10	42.30	95.54	1.90	Clostridiales	Firmicutes	Ruminococcus sp. AF46-10NS
Sample_B11_2.bin_5	B112.M005	22	2.35	199.71	41.87	42.12	99.32	0.00	Clostridiales	Firmicutes	Coprococcus sp. OM04-5BH
Sample_A23_1.bin_5	A231.M005	25	2.02	128.73	60.09	42.08	92.61	0.67	Clostridiales	Firmicutes	Firmicutes bacterium CAG:83
Sample_A04_3.bin_5	A043.M005	73	2.13	37.57	33.62	41.29	97.30	0.71	Bacteria	Firmicutes	Holdemanella biformis
Sample_A02_3.bin_5	A023.M005	21	2.30	145.02	44.35	41.07	97.09	0.00	Clostridiales	Firmicutes	Ruminococcus sp. CAG:563
Sample_A18_1.bin_5	A181.M005	42	1.59	51.67	37.59	40.90	94.33	1.69	Lactobacillales	Firmicutes	Streptococcus gallolyticus
Sample_A06_1.bin_5	A061.M005	32	2.68	118.35	44.98	40.48	93.67	0.34	Clostridiales	Firmicutes	Ruminococcus sp. AF20-12LB

Sample_A19_1.bin_5	A191.M005	70	2.03	34.68	58.91	40.25	90.67	3.23	Clostridiales	Firmicutes	Faecalibacterium prausnitzii
Sample_B24_3.bin_5	B243.M005	21	1.62	85.09	43.54	40.19	99.43	0.07	Leuconostocaceae	Firmicutes	Leuconostoc lactis
Sample_A22_1.bin_5	A221.M005	25	1.01	72.44	26.13	39.80	84.83	1.69	Bacteria	Firmicutes	Clostridium sp. CAG:628
Sample_A30_2.bin_5	A302.M005	45	2.21	86.44	53.07	39.55	98.38	0.81	Clostridia	Firmicutes	Clostridium sp. CAG:226
Sample_A09_1.bin_6	A091.M006	49	2.65	73.30	56.15	39.36	89.03	0.67	Clostridiales	Firmicutes	Firmicutes bacterium CAG:124
Sample_B17_2.bin_6	B172.M006	42	4.01	133.01	52.30	39.12	85.26	1.73	Enterobacteriaceae	Proteobacteria	Citrobacter portucalensis
Sample_A31_2.bin_6	A312.M006	11	3.29	494.09	43.65	39.11	99.62	0.00	Bacteroidales	Bacteroidetes	Bacteroides sp. CAG:20
Sample_B20_2.bin_6	B202.M006	81	2.24	54.95	59.95	39.03	100.00	2.42	Actinobacteria	Actinobacteria	Collinsella aerofaciens
Sample_B13_1.bin_6	B131.M006	17	2.20	214.97	45.47	39.01	97.87	0.71	Clostridiales	Firmicutes	Firmicutes bacterium CAG:145
Sample_B13_2.bin_6	B132.M006	31	2.54	153.48	37.55	38.94	98.25	3.67	Clostridiales	Firmicutes	Eubacterium sp.
Sample_A29_2.bin_6	A292.M006	39	3.11	129.03	62.21	38.75	92.75	0.13	Clostridiales	Firmicutes	Flavonifractor plautii
Sample_B12_2.bin_6	B122.M006	27	2.24	147.51	59.86	38.57	100.00	0.81	Actinobacteria	Actinobacteria	Collinsella aerofaciens
Sample_A36_3.bin_6	A363.M006	11	1.60	295.31	48.64	38.33	93.95	0.81	Clostridia	Firmicutes	Subdoligranulum sp. CAG:314
Sample_B05_2.bin_6	B052.M006	70	2.84	48.18	41.69	38.28	90.92	0.00	Clostridiales	Firmicutes	Blautia obeum
Sample_A33_3.bin_6	A333.M006	17	2.59	226.38	35.91	37.69	97.98	0.00	Clostridiales	Firmicutes	Eubacterium sp. CAG:76

Sample_A09_3.bin_6	A093.M006	57	2.77	66.00	46.87	37.46	92.75	1.87	Lachnospiraceae	Firmicutes	Lachnospiraceae bacterium
Sample_A01_2.bin_6	A012.M006	38	2.55	136.35	45.06	37.24	98.99	0.00	Clostridiales	Firmicutes	Eubacterium siraeum
Sample_B22_3.bin_6	B223.M006	35	2.84	127.03	48.29	37.24	97.67	0.63	Clostridiales	Firmicutes	Fusicatenibacter saccharivorans
Sample_A21_3.bin_6	A213.M006	64	2.71	52.82	46.32	37.15	90.29	0.06	Bacteroidales	Bacteroidetes	Bacteroides coprophilus CAG:333
Sample_B20_1.bin_6	B201.M006	22	4.69	337.29	56.05	36.89	99.37	0.04	Enterobacteriaceae	Proteobacteria	Enterobacter bugandensis
Sample_B11_2.bin_6	B112.M006	29	2.81	148.12	51.72	36.68	92.61	1.34	Clostridiales	Firmicutes	Ruminococcaceae bacterium
Sample_B21_3.bin_6	B213.M006	54	1.27	50.88	34.63	36.41	99.37	0.63	Lactobacillales	Firmicutes	Lactobacillus sanfranciscensis
Sample_A33_1.bin_6	A331.M006	38	2.52	109.83	41.50	36.10	98.42	0.00	Lachnospiraceae	Firmicutes	Dorea longicatena
Sample_B13_2.bin_7	B132.M007	44	1.67	58.54	53.62	36.09	96.62	0.19	Bacteria	Elusimicrobia	Elusimicrobium sp. An273
Sample_A30_3.bin_7	A303.M007	52	3.15	82.43	44.77	35.90	93.80	0.00	Bacteroidales	Bacteroidetes	Bacteroides plebeius
Sample_B12_2.bin_7	B122.M007	13	1.86	246.11	44.59	35.81	97.98	0.34	Clostridiales	Firmicutes	Eubacterium sp. CAG:180
Sample_A18_1.bin_7	A181.M007	17	2.71	225.13	44.17	35.67	98.65	0.00	Clostridiales	Firmicutes	Lachnospiraceae bacterium
Sample_A17_3.bin_7	A173.M007	35	2.81	105.06	52.81	35.26	98.73	0.00	Clostridiales	Firmicutes	Clostridium sp. CAG:58
Sample_A02_3.bin_7	A023.M007	64	3.35	80.16	53.84	34.95	91.14	0.00	Bacteroidales	Bacteroidetes	Bacteroides sp. CAG:462
Sample_A27_3.bin_7	A273.M007	81	2.10	42.46	53.89	34.89	86.24	0.67	Clostridiales	Firmicutes	Ruminococcus sp. CAG:330

Sample_B05_2.bin_7	B052.M007	47	1.33	44.25	26.91	34.33	93.82	0.00	Bacteria	Firmicutes	Clostridium sp. CAG:710
Sample_B15_3.bin_7	B153.M007	61	2.35	54.83	44.79	34.01	98.18	0.63	Clostridiales	Firmicutes	uncultured Ruminococcus sp.
Sample_B11_2.bin_7	B112.M007	22	2.12	136.47	37.49	33.28	99.32	0.00	Clostridiales	Firmicutes	Butyrivibrio sp.
Sample_B14_3.bin_7	B143.M007	6	1.94	358.82	30.48	32.72	88.88	0.85	Bacteria	Firmicutes	Clostridium sp. CAG:768
Sample_A32_2.bin_7	A322.M007	32	2.08	84.05	47.56	32.70	97.98	0.00	Clostridiales	Firmicutes	Anaerotignum faecicola
Sample_B16_3.bin_7	B163.M007	46	2.24	62.57	54.15	32.58	100.00	0.00	Selenomonadales	Firmicutes	Megasphaera sp. NM10
Sample_A06_2.bin_7	A062.M007	75	2.47	63.68	46.40	32.22	94.63	1.34	Clostridiales	Firmicutes	Ruminococcaceae bacterium
Sample_A04_2.bin_7	A042.M007	52	3.24	83.90	30.94	31.69	97.16	0.00	Bacteria	Firmicutes	Coprobacillus cateniformis
Sample_A24_3.bin_7	A243.M007	77	2.95	73.93	29.25	31.44	100.00	2.25	Bacteria	Fusobacteria	Fusobacterium varium
Sample_A03_1.bin_7	A031.M007	42	3.54	119.02	37.92	31.36	92.61	2.01	Clostridiales	Firmicutes	Lachnospiraceae bacterium
Sample_B24_1.bin_7	B241.M007	30	2.77	144.32	43.05	31.34	99.41	0.00	Lachnospiraceae	Firmicutes	Ruminococcus gnavus
Sample_A27_1.bin_7	A271.M007	59	2.42	51.33	43.33	31.13	87.34	0.42	Clostridiales	Firmicutes	Ruminococcus sp. AF41-9
Sample_A03_2.bin_7	A032.M007	44	2.02	62.79	55.52	31.07	81.69	0.38	Bacteroidales	Bacteroidetes	Muribaculaceae bacterium Isolate-013 (NCI)
Sample_B16_2.bin_7	B162.M007	78	2.12	39.85	34.19	30.77	93.66	0.37	Bacteria	Firmicutes	Eubacterium sp. AM28-29
Sample_A24_1.bin_7	A241.M007	79	2.67	49.09	47.92	30.10	95.28	0.13	Bacteroidales	Bacteroidetes	Porphyromonadaceae bacterium

Sample_B02_2.bin_8	B022.M008	46	6.31	163.97	43.54	30.02	98.46	0.77	Bacteroidales	Bacteroidetes	Parabacteroides goldsteinii
Sample_A18_2.bin_8	A182.M008	27	2.71	120.47	43.76	29.87	94.92	0.48	Lachnospiraceae	Firmicutes	Roseburia faecis
Sample_B21_1.bin_8	B211.M008	71	2.16	64.61	48.50	29.77	95.96	0.81	Clostridia	Firmicutes	Clostridia bacterium
Sample_A33_1.bin_8	A331.M008	49	2.54	94.95	41.36	29.74	98.83	0.00	Lachnospiraceae	Firmicutes	uncultured Ruminococcus sp.
Sample_B16_3.bin_8	B163.M008	91	3.00	50.10	58.22	29.62	100.00	1.01	Clostridia	Firmicutes	Clostridiales bacterium
Sample_A36_2.bin_8	A362.M008	37	2.68	105.98	44.09	29.34	95.65	1.93	Lachnospiraceae	Firmicutes	Lachnospiraceae bacterium
Sample_B17_1.bin_8	B171.M008	57	1.98	43.66	43.28	29.24	87.15	0.00	Lachnospiraceae	Firmicutes	Ruminococcus lactaris
Sample_A28_2.bin_8	A282.M008	32	1.17	54.71	26.72	28.99	90.16	1.69	Bacteria	Firmicutes	Firmicutes bacterium CAG:321
Sample_A30_2.bin_8	A302.M008	20	3.87	336.81	43.46	28.54	82.25	0.00	Bacteroidales	Bacteroidetes	Bacteroides fragilis
Sample_A29_2.bin_8	A292.M008	44	2.97	103.40	45.22	28.45	86.94	0.95	Clostridiales	Firmicutes	uncultured Blautia sp.
Sample_B15_2.bin_8	B152.M008	23	2.18	151.87	36.17	28.34	96.42	0.00	Lactobacillus	Firmicutes	Lactobacillus crispatus
Sample_A28_3.bin_8	A283.M008	21	2.20	156.67	37.11	28.33	99.32	0.34	Clostridiales	Firmicutes	uncultured Ruminococcus sp.
Sample_A21_1.bin_8	A211.M008	21	2.96	297.89	43.77	28.05	99.32	0.00	Clostridiales	Firmicutes	Coprococcus sp. BIOML-A1
Sample_B14_3.bin_8	B143.M008	64	3.05	65.57	29.01	27.96	99.05	0.00	Bacteria	Firmicutes	uncultured Clostridium sp.
Sample_A06_1.bin_8	A061.M008	32	2.50	113.71	33.18	27.67	99.32	0.00	Clostridiales	Firmicutes	uncultured Eubacterium sp.

Sample_A02_2.bin_8	A022.M008	28	1.85	88.39	32.58	27.41	99.47	0.00	Lactobacillales	Firmicutes	Lactobacillus salivarius
Sample_B18_1.bin_9	B181.M009	41	2.76	83.65	49.09	27.24	97.46	2.53	Clostridiales	Firmicutes	Lachnoclostridium sp.
Sample_A36_3.bin_9	A363.M009	74	2.41	47.93	61.75	27.19	97.37	1.25	Proteobacteria	Proteobacteria	Sutterella megalosphaeroides
Sample_B05_3.bin_9	B053.M009	27	2.12	180.56	35.17	27.19	87.17	0.28	Bacteria	Candidatus Melainabacteria	Candidatus Gastranaerophilales bacterium HUM_10
Sample_B15_2.bin_9	B152.M009	98	3.20	54.90	56.12	26.96	98.38	1.61	Clostridia	Firmicutes	Clostridiales bacterium
Sample_A03_1.bin_9	A031.M009	79	2.22	36.64	38.55	26.93	89.93	0.67	Clostridiales	Firmicutes	uncultured Eubacterium sp.
Sample_A19_1.bin_9	A191.M009	21	2.34	158.59	33.04	26.81	98.12	0.67	Clostridiales	Firmicutes	Eubacterium sp. CAG:274
Sample_A12_2.bin_9	A122.M009	32	2.20	173.60	46.08	26.73	97.98	0.00	Clostridiales	Firmicutes	Ruminococcus sp.
Sample_A09_2.bin_9	A092.M009	25	2.52	138.36	51.47	26.68	95.30	0.00	Clostridiales	Firmicutes	Ruminococcus sp. OM06-36AC
Sample_B23_3.bin_9	B233.M009	33	2.06	84.56	58.84	26.31	97.98	0.00	Clostridiales	Firmicutes	Oscillibacter sp.
Sample_A30_1.bin_9	A301.M009	48	1.75	66.81	38.91	26.04	98.58	0.15	Streptococcus	Firmicutes	Streptococcus thermophilus
Sample_B02_1.bin_9	B021.M009	29	5.51	259.88	49.47	25.94	94.90	0.00	Clostridiales	Firmicutes	Enterocloster bolteae
Sample_A06_3.bin_9	A063.M009	31	1.90	83.47	56.93	25.87	86.57	0.00	Clostridiales	Firmicutes	Clostridiales bacterium
Sample_A04_3.bin_9	A043.M009	29	2.57	156.09	36.34	25.81	98.65	0.00	Clostridiales	Firmicutes	Eubacterium sp. CAG:86
Sample_B16_2.bin_9	B162.M009	11	1.71	189.57	47.12	25.63	86.57	0.00	Clostridiales	Firmicutes	Ruminococcus sp. CAG:403

Sample_A29_3.bin_9	A293.M009	19	1.72	164.09	45.16	25.61	92.17	0.67	Clostridiales	Firmicutes	Eubacterium sp. CAG:841
Sample_A21_2.bin_10	A212.M010	39	2.65	97.88	42.37	25.51	91.27	0.00	Clostridiales	Firmicutes	Roseburia sp. CAG:309
Sample_B15_3.bin_10	B153.M010	40	2.39	100.34	34.28	25.43	99.32	0.00	Clostridiales	Firmicutes	Eubacterium ventriosum
Sample_A18_3.bin_10	A183.M010	29	2.05	98.27	57.81	25.40	96.64	0.00	Clostridiales	Firmicutes	Oscillibacter sp. 57_20
Sample_B03_3.bin_10	B033.M010	16	1.98	227.64	45.28	25.37	99.40	1.20	Selenomonadales	Firmicutes	Phascolarctobacterium sp. CAG:266
Sample_A01_2.bin_10	A012.M010	25	2.23	160.82	33.27	25.33	99.32	0.00	Clostridiales	Firmicutes	Eubacterium sp. AF36-5BH
Sample_A03_2.bin_10	A032.M010	48	1.86	48.17	59.64	25.31	81.50	0.17	Clostridiales	Firmicutes	Subdoligranulum sp. 60_17
Sample_A09_1.bin_10	A091.M010	34	2.10	122.40	48.47	25.25	81.71	0.54	Rhodospirillales	Proteobacteria	Alphaproteobacteria bacterium
Sample_A23_3.bin_10	A233.M010	45	2.24	83.24	60.72	25.16	94.55	0.00	Clostridiales	Firmicutes	Subdoligranulum sp. 60_17
Sample_B21_2.bin_10	B212.M010	62	2.37	55.24	57.40	24.91	96.87	0.00	Proteobacteria	Proteobacteria	Mesosutterella multiformis
Sample_A07_2.bin_10	A072.M010	101	2.25	39.41	44.15	24.90	99.38	1.05	Selenomonadales	Firmicutes	Phascolarctobacterium faecium
Sample_A23_2.bin_10	A232.M010	45	3.28	127.17	58.21	24.74	97.95	0.00	Clostridiales	Firmicutes	Ruthenibacterium lactatiformans
Sample_A18_2.bin_10	A182.M010	32	1.95	115.82	59.54	24.69	100.00	0.81	Actinobacteria	Actinobacteria	Collinsella sp. WCA1-178-WT-3 (M2)
Sample_A33_1.bin_10	A331.M010	114	4.70	65.02	55.76	24.65	98.09	4.63	Enterobacteriaceae	Proteobacteria	Enterobacter cloacae
Sample_B02_3.bin_10	B023.M010	27	2.50	99.14	41.20	24.55	98.06	1.22	Lachnospiraceae	Firmicutes	Roseburia sp. AF15-21

Sample_B02_1.bin_10	B021.M010	62	2.38	60.41	62.04	24.42	95.65	0.62	Proteobacteria	Proteobacteria	Sutterella megalosphaeroides
Sample_A03_1.bin_10	A031.M010	37	1.01	34.97	24.69	24.25	91.57	2.41	Bacteria	Tenericutes	Mycoplasma sp. CAG:611
Sample_A27_2.bin_10	A272.M010	107	2.72	34.62	41.83	24.03	91.83	1.34	Lachnospiraceae	Firmicutes	Lachnospiraceae bacterium
Sample_A31_2.bin_10	A312.M010	24	2.24	126.51	52.17	23.99	97.27	0.00	Clostridiales	Firmicutes	Ruminococcaceae bacterium
Sample_A19_2.bin_10	A192.M010	18	2.53	249.13	41.33	23.85	99.32	0.00	Clostridiales	Firmicutes	uncultured Coprococcus sp.
Sample_A18_2.bin_11	A182.M011	23	2.09	178.31	55.88	23.72	98.75	0.63	Proteobacteria	Proteobacteria	Sutterella sp. AM11-39
Sample_A36_3.bin_11	A363.M011	57	3.04	75.89	46.05	23.69	97.12	1.63	Lachnospiraceae	Firmicutes	Lachnospiraceae bacterium
Sample_B22_2.bin_11	B222.M011	43	2.84	93.91	49.17	23.67	99.36	0.00	Clostridiales	Firmicutes	Clostridium sp. CAG:299
Sample_B16_3.bin_11	B163.M011	20	1.87	112.22	62.40	23.66	87.91	0.17	Clostridiales	Firmicutes	Clostridiales bacterium
Sample_B17_3.bin_11	B173.M011	55	2.83	81.77	55.22	23.61	100.00	0.00	Proteobacteria	Proteobacteria	Sutterella wadsworthensis
Sample_A31_2.bin_11	A312.M011	62	3.43	74.21	41.02	23.51	97.46	2.22	Clostridiales	Firmicutes	uncultured Lachnospira sp.
Sample_A01_3.bin_11	A013.M011	44	2.35	81.55	60.17	23.49	100.00	0.00	Bacteria	Synergistetes	Pyramidobacter piscolens
Sample_B15_2.bin_11	B152.M011	34	1.82	72.88	38.75	23.47	90.16	1.09	Lactobacillales	Firmicutes	Lactobacillus reuteri
Sample_B11_3.bin_11	B113.M011	29	2.43	101.93	45.05	23.34	95.46	0.00	Lachnospiraceae	Firmicutes	Roseburia sp. CAG:197
Sample_A29_1.bin_11	A291.M011	50	2.41	74.96	41.88	23.25	96.42	0.67	Clostridiales	Firmicutes	Clostridiales bacterium

Sample_A22_1.bin_11	A221.M011	49	2.50	72.17	42.27	23.22	98.65	0.00	Clostridiales	Firmicutes	Clostridium sp. OM05-6BH
Sample_A03_2.bin_11	A032.M011	36	2.21	128.55	54.47	23.16	98.12	0.63	Proteobacteria	Proteobacteria	Sutterella sp. AM11-39
Sample_A30_1.bin_12	A301.M012	27	1.22	108.21	26.13	23.03	90.66	1.33	Bacteria	Firmicutes	Firmicutes bacterium CAG:321
Sample_B21_2.bin_12	B212.M012	28	2.60	132.69	40.67	22.93	98.65	0.00	Clostridiales	Firmicutes	Lachnospiraceae bacterium
Sample_A23_2.bin_12	A232.M012	14	2.04	214.26	40.69	22.87	99.32	0.00	Clostridiales	Firmicutes	Ruminococcus bromii
Sample_A27_2.bin_12	A272.M012	13	2.05	181.00	59.58	22.83	99.54	0.00	Bifidobacteriaceae	Actinobacteria	Bifidobacterium adolescentis
Sample_B05_2.bin_12	B052.M012	19	2.68	185.02	55.01	22.83	100.00	0.00	Bacteroidetes	Bacteroidetes	Alistipes sp. AF17-16
Sample_A02_3.bin_12	A023.M012	22	2.46	149.02	42.32	22.72	95.30	0.00	Clostridiales	Firmicutes	Bacteroides pectinophilus
Sample_B17_3.bin_12	B173.M012	54	2.23	62.99	60.93	22.58	89.26	1.34	Clostridiales	Firmicutes	uncultured Flavonifractor sp.
Sample_A23_3.bin_12	A233.M012	23	1.99	150.20	39.38	22.50	98.97	0.00	Pasteurellaceae	Proteobacteria	Haemophilus parainfluenzae
Sample_B20_3.bin_12	B203.M012	15	1.94	175.13	36.69	22.45	98.12	0.00	Lactobacillales	Firmicutes	Streptococcus mutans
Sample_B15_1.bin_12	B151.M012	42	2.49	120.13	57.48	22.44	95.30	0.00	Clostridiales	Firmicutes	Ruminococcaceae bacterium
Sample_B05_3.bin_12	B053.M012	41	2.57	85.57	38.58	22.44	91.54	0.00	Lachnospiraceae	Firmicutes	Eubacterium rectale
Sample_A31_2.bin_12	A312.M012	64	2.12	61.92	33.74	22.35	98.65	0.34	Clostridiales	Firmicutes	Eubacterium sp. CAG:581
Sample_A29_1.bin_12	A291.M012	59	2.51	86.61	54.89	22.22	98.65	0.67	Clostridiales	Firmicutes	uncultured Butyricicoccus sp.

Sample_B18_2.bin_12	B182.M012	69	4.82	106.19	45.08	22.19	99.42	0.00	Bacteroidales	Bacteroidetes	Parabacteroides distasonis
Sample_A09_2.bin_13	A092.M013	35	2.20	103.86	59.90	22.19	100.00	0.81	Actinobacteria	Actinobacteria	Collinsella aerofaciens
Sample_B14_3.bin_13	B143.M013	52	2.19	63.16	60.34	22.11	88.30	0.27	Clostridia	Firmicutes	Clostridiales bacterium
Sample_A28_2.bin_13	A282.M013	74	1.96	43.70	48.22	22.09	94.63	0.02	Clostridiales	Firmicutes	Ruminococcaceae bacterium
Sample_A24_2.bin_13	A242.M013	37	2.97	122.70	49.41	22.08	95.17	0.37	Bacteroidales	Bacteroidetes	Prevotella sp. AG:487_50_53
Sample_A23_3.bin_13	A233.M013	29	2.12	94.55	61.25	22.02	96.30	0.67	Clostridiales	Firmicutes	Clostridiales bacterium
Sample_B17_1.bin_13	B171.M013	52	2.67	66.70	36.92	21.99	98.65	1.34	Clostridiales	Firmicutes	Anaerostipes hadrus
Sample_B18_1.bin_13	B181.M013	53	1.74	40.74	57.44	21.96	84.91	0.00	Clostridiales	Firmicutes	Clostridiales bacterium
Sample_A18_2.bin_13	A182.M013	38	1.92	75.76	33.47	21.93	97.09	0.00	Bacilli	Firmicutes	Melissococcus sp. OM08-11BH
Sample_A27_2.bin_13	A272.M013	10	1.76	186.44	53.98	21.86	100.00	0.12	Actinobacteria	Actinobacteria	Eggerthella sp. CAG:298
Sample_B22_1.bin_13	B221.M013	46	2.52	90.72	45.86	21.72	95.30	0.67	Clostridiales	Firmicutes	Ruminococcaceae bacterium
Sample_B22_2.bin_13	B222.M013	10	2.01	229.73	38.57	21.69	98.20	0.00	Selenomonadales	Firmicutes	Veillonella parvula
Sample_A19_2.bin_14	A192.M014	91	2.43	43.12	52.33	21.63	100.00	0.00	Firmicutes	Firmicutes	Dialister succinatiphilus
Sample_B21_3.bin_14	B213.M014	12	2.42	289.44	58.52	21.63	98.39	0.00	Bacteroidetes	Bacteroidetes	Alistipes sp. AF17-16
Sample_A02_1.bin_14	A021.M014	21	2.30	174.23	56.30	21.53	94.63	0.67	Clostridiales	Firmicutes	Ruminococcaceae bacterium

Sample_B23_3.bin_14	B233.M014	35	3.90	142.43	45.50	21.18	99.61	0.00	Bacteroidales	Bacteroidetes	Parabacteroides merdae
Sample_B20_3.bin_14	B203.M014	30	2.16	159.43	59.73	20.93	100.00	0.81	Actinobacteria	Actinobacteria	Collinsella aerofaciens
Sample_A06_3.bin_14	A063.M014	38	2.64	90.51	38.52	20.92	97.77	0.00	Clostridiales	Firmicutes	Firmicutes bacterium AM10-47
Sample_A22_1.bin_14	A221.M014	56	2.80	65.01	46.75	20.89	96.13	1.93	Lachnospiraceae	Firmicutes	uncultured Clostridium sp.
Sample_A02_3.bin_14	A023.M014	26	2.57	111.00	45.16	20.85	88.25	0.00	Clostridiales	Firmicutes	Clostridium sp. OM07-9AC
Sample_A28_3.bin_14	A283.M014	62	2.74	66.24	38.24	20.67	97.98	1.01	Clostridiales	Firmicutes	uncultured Clostridium sp.
Sample_A23_3.bin_14	A233.M014	42	2.73	87.50	57.37	20.66	97.76	2.68	Clostridiales	Firmicutes	Firmicutes bacterium CAG:137
Sample_A24_1.bin_14	A241.M014	27	1.64	100.58	34.70	20.64	91.77	1.08	Bacteria	Firmicutes	Faecalitalea cylindroides
Sample_A30_2.bin_14	A302.M014	51	3.17	82.69	42.93	20.52	93.71	0.12	Lachnospiraceae	Firmicutes	Roseburia intestinalis
Sample_A36_1.bin_14	A361.M014	28	3.14	177.69	57.85	20.48	97.95	1.36	Bacteria	Verrucomicrobia	Akkermansia muciniphila
Sample_B02_2.bin_15	B022.M015	182	1.96	14.46	53.62	20.37	86.12	0.00	Clostridiales	Firmicutes	Ruminococcaceae bacterium
Sample_A33_2.bin_15	A332.M015	9	2.68	360.40	55.03	20.25	100.00	0.00	Bacteroidetes	Bacteroidetes	Alistipes indistinctus
Sample_B22_2.bin_15	B222.M015	53	1.13	28.47	24.37	20.11	84.83	4.09	Bacteria	Firmicutes	Firmicutes bacterium
Sample_A23_3.bin_15	A233.M015	26	2.54	130.81	38.89	20.11	87.24	0.00	Clostridiales	Firmicutes	Clostridium sp. CAG:230
Sample_A32_2.bin_15	A322.M015	29	2.50	146.63	58.56	20.05	96.33	1.38	Deltaproteobacteria	Proteobacteria	Desulfovibrio sp.

Sample_A07_3.bin_15	A073.M015	81	3.01	55.94	44.17	20.01	95.22	0.00	Clostridiales	Firmicutes	Clostridium sp. TM06-18
Sample_A22_3.bin_15	A223.M015	79	3.90	73.44	43.44	19.75	98.63	0.00	Bacteria	Bacteroidetes	Odoribacter splanchnicus
Sample_A05_1.bin_15	A051.M015	32	1.73	95.51	60.00	19.74	88.59	0.67	Clostridiales	Firmicutes	Clostridia bacterium
Sample_A22_2.bin_15	A222.M015	19	2.09	170.35	33.54	19.57	95.30	1.34	Clostridiales	Firmicutes	Eubacterium sp. CAG:274
Sample_B22_3.bin_15	B223.M015	38	2.10	80.48	57.68	19.47	95.93	2.35	Clostridiales	Firmicutes	Faecalibacterium prausnitzii
Sample_B06_1.bin_15	B061.M015	45	2.89	84.95	37.02	19.47	98.48	0.00	Clostridiales	Firmicutes	Eubacterium sp. CAG:252
Sample_A27_2.bin_15	A272.M015	56	2.25	76.94	61.10	19.45	91.27	0.31	Clostridiales	Firmicutes	Clostridiales bacterium
Sample_B22_1.bin_15	B221.M015	61	2.56	64.00	58.38	19.43	95.79	1.36	Clostridiales	Firmicutes	Ruminococcaceae bacterium
Sample_A01_1.bin_15	A011.M015	64	2.91	71.70	46.14	19.40	93.98	0.32	Clostridiales	Firmicutes	Lachnospiraceae bacterium AM48-27BH
Sample_A17_1.bin_15	A171.M015	20	1.73	95.77	61.33	19.36	85.57	0.00	Clostridiales	Firmicutes	Oscillibacter sp. CAG:155
Sample_A01_2.bin_15	A012.M015	57	1.90	47.24	62.16	19.26	99.37	0.63	Proteobacteria	Proteobacteria	Sutterella sp. AM11-39
Sample_B18_1.bin_15	B181.M015	65	2.14	42.79	58.86	19.22	91.22	0.00	Clostridiales	Firmicutes	Subdoligranulum variabile
Sample_A18_1.bin_16	A181.M016	31	2.58	105.96	43.76	19.01	92.61	0.67	Clostridiales	Firmicutes	Coprococcus catus
Sample_B02_2.bin_16	B022.M016	56	2.27	64.58	56.97	18.95	96.42	0.67	Clostridiales	Firmicutes	Firmicutes bacterium CAG:110
Sample_A33_3.bin_16	A333.M016	48	2.60	80.59	46.13	18.84	94.93	0.63	Clostridiales	Firmicutes	Blautia sp. KGMB01111

Sample_A31_1.bin_16	A311.M016	52	2.30	56.53	44.98	18.67	87.34	1.27	Clostridiales	Firmicutes	uncultured Blautia sp.
Sample_A01_3.bin_16	A013.M016	48	2.59	82.87	37.73	18.64	95.97	1.34	Clostridiales	Firmicutes	uncultured Eubacterium sp.
Sample_A02_3.bin_16	A023.M016	24	2.48	149.26	42.91	18.56	99.31	0.00	Clostridiales	Firmicutes	uncultured Ruminococcus sp.
Sample_A03_2.bin_16	A032.M016	37	2.10	76.20	36.70	18.51	89.93	1.51	Clostridiales	Firmicutes	Anaerostipes hadrus
Sample_A04_3.bin_16	A043.M016	42	2.78	90.69	46.18	18.47	97.98	1.65	Clostridiales	Firmicutes	Lachnospiraceae bacterium
Sample_A22_2.bin_16	A222.M016	26	1.99	94.77	49.04	18.39	99.26	0.18	Bacteria	Bacteroidetes	Flavobacteriales bacterium
Sample_A06_1.bin_16	A061.M016	33	2.48	95.12	42.68	18.27	94.30	0.02	Clostridiales	Firmicutes	uncultured Blautia sp.
Sample_A01_2.bin_16	A012.M016	26	2.76	223.84	41.83	18.17	98.65	0.00	Clostridiales	Firmicutes	Eubacterium sp. CAG:38
Sample_B21_3.bin_17	B213.M017	27	2.08	90.80	41.75	18.13	91.57	1.11	Lachnospiraceae	Firmicutes	Lachnospiraceae bacterium
Sample_A31_3.bin_17	A313.M017	49	2.48	60.73	59.26	17.98	97.27	0.00	Clostridiales	Firmicutes	Subdoligranulum sp. 60_17
Sample_A27_3.bin_17	A273.M017	35	2.06	99.33	61.28	17.97	91.61	0.67	Clostridiales	Firmicutes	Firmicutes bacterium CAG:176_63_11
Sample_A01_2.bin_17	A012.M017	56	2.53	64.40	29.13	17.93	98.87	1.12	Bacteria	Fusobacteria	Fusobacterium mortiferum
Sample_A31_1.bin_17	A311.M017	36	1.74	67.38	61.42	17.86	95.80	0.67	Clostridiales	Firmicutes	Firmicutes bacterium CAG:129
Sample_A09_2.bin_17	A092.M017	101	3.07	54.14	52.33	17.84	97.31	0.00	Clostridiales	Firmicutes	Eubacterium sp. CAG:115
Sample_B16_2.bin_17	B162.M017	47	3.43	105.94	71.62	17.81	99.48	1.38	Actinomycetales	Actinobacteria	Aeromicrobium sp. Leaf289

Sample_A31_2.bin_17	A312.M017	67	2.89	51.79	48.88	17.72	99.36	0.00	Clostridiales	Firmicutes	uncultured Clostridium sp.
Sample_A29_1.bin_17	A291.M017	29	2.22	115.97	40.62	17.69	91.13	0.00	Clostridiales	Firmicutes	Tyzzerella nexilis
Sample_A24_1.bin_17	A241.M017	188	5.59	44.16	59.72	17.67	93.91	4.05	Bacteria	Lentisphaerae	Victivallis vadensis
Sample_A02_3.bin_18	A023.M018	20	1.99	142.24	38.39	17.64	95.97	0.00	Clostridiales	Firmicutes	Anaeromassilibacillus sp. An250
Sample_A18_2.bin_18	A182.M018	58	2.40	52.94	56.76	17.64	99.10	0.67	Clostridiales	Firmicutes	Clostridiales bacterium
Sample_A31_3.bin_18	A313.M018	29	1.09	55.14	26.74	17.60	86.51	0.00	Bacteria	Firmicutes	Acholeplasma sp. CAG:878
Sample_B02_2.bin_18	B022.M018	119	4.35	48.05	47.92	17.53	82.73	1.27	Clostridiales	Firmicutes	uncultured Clostridium sp.
Sample_A03_2.bin_18	A032.M018	39	2.09	81.30	58.29	17.41	94.40	0.00	Clostridiales	Firmicutes	Clostridiales bacterium
Sample_A29_1.bin_18	A291.M018	37	2.15	97.09	60.05	17.39	89.93	0.00	Clostridiales	Firmicutes	Clostridiales bacterium
Sample_A06_3.bin_18	A063.M018	108	3.27	44.11	38.53	17.29	97.35	0.00	Bacteroidales	Bacteroidetes	Coprobacter fastidiosus
Sample_A22_1.bin_18	A221.M018	161	4.20	41.11	59.95	17.07	100.00	0.00	Deltaproteobacteria	Proteobacteria	Bilophila wadsworthia
Sample_B22_2.bin_18	B222.M018	91	2.17	36.67	60.02	16.93	98.09	2.42	Actinobacteria	Actinobacteria	Collinsella aerofaciens
Sample_A27_1.bin_18	A271.M018	36	2.11	108.24	51.92	16.71	98.65	0.67	Clostridiales	Firmicutes	Clostridium sp. CAG:169
Sample_B21_2.bin_18	B212.M018	78	2.11	44.88	60.06	16.57	96.77	0.81	Actinobacteria	Actinobacteria	Collinsella aerofaciens
Sample_B16_3.bin_19	B163.M019	142	1.89	17.45	33.89	16.57	99.05	0.00	Bacteria	Firmicutes	Catenibacterium sp. AM22-15

Sample_B21_3.bin_19	B213.M019	51	5.13	167.20	42.61	16.50	98.37	0.11	Bacteria	Bacteroidetes	Odoribacter sp. AF21-41
Sample_A19_2.bin_19	A192.M019	30	2.35	141.43	46.37	16.49	100.00	0.48	Bacteroidetes	Bacteroidetes	Alistipes indistinctus
Sample_A03_2.bin_19	A032.M019	50	2.08	96.88	60.10	16.46	99.19	2.42	Actinobacteria	Actinobacteria	Collinsella sp. AM34-10
Sample_A36_3.bin_19	A363.M019	27	2.41	119.30	42.22	16.44	87.81	0.63	Clostridiales	Firmicutes	Blautia obeum
Sample_A27_2.bin_19	A272.M019	5	1.21	621.57	27.73	16.37	93.25	1.28	Bacteria	Firmicutes	Clostridium sp. CAG:417
Sample_B15_3.bin_19	B153.M019	44	2.86	99.70	37.34	16.33	97.98	0.00	Clostridiales	Firmicutes	Lachnospira eligens
Sample_A04_3.bin_19	A043.M019	55	3.00	76.49	41.08	16.30	97.42	0.00	Clostridiales	Firmicutes	Eubacterium sp.
Sample_A02_2.bin_19	A022.M019	26	1.90	125.91	47.90	16.24	94.63	0.00	Clostridiales	Firmicutes	Ruminococcaceae bacterium
Sample_A18_2.bin_19	A182.M019	68	2.71	51.44	58.54	16.14	90.72	1.08	Clostridia	Firmicutes	Clostridiales bacterium
Sample_A07_2.bin_19	A072.M019	64	1.98	60.49	39.48	15.93	98.50	0.88	Pasteurellaceae	Proteobacteria	Haemophilus parainfluenzae
Sample_A01_2.bin_19	A012.M019	50	2.25	57.24	58.90	15.88	90.41	1.34	Clostridiales	Firmicutes	Firmicutes bacterium CAG:170
Sample_A23_2.bin_20	A232.M020	12	1.93	196.86	45.41	15.86	88.70	0.12	Actinobacteria	Actinobacteria	Cryptobacterium sp. CAG:338
Sample_B16_1.bin_20	B161.M020	109	2.25	26.65	48.40	15.81	80.23	0.00	Bacteroidales	Bacteroidetes	Prevotella sp. CAG:891
Sample_B16_2.bin_20	B162.M020	43	1.98	78.90	38.98	15.74	100.00	0.00	Selenomonadales	Firmicutes	Veillonella atypica
Sample_B21_3.bin_20	B213.M020	80	2.26	46.54	60.02	15.65	100.00	0.81	Actinobacteria	Actinobacteria	Collinsella aerofaciens

Sample_A18_3.bin_20	A183.M020	169	2.44	26.23	49.92	15.61	85.13	1.90	Clostridiales	Firmicutes	Clostridium sp. AM33-3
Sample_A07_2.bin_20	A072.M020	75	2.64	46.25	26.79	15.57	93.99	0.00	Clostridiales	Firmicutes	uncultured Clostridium sp.
Sample_A18_2.bin_20	A182.M020	84	2.60	55.76	63.75	15.53	98.12	0.89	Deltaproteobacteria	Proteobacteria	Desulfovibrio piger
Sample_A33_3.bin_20	A333.M020	94	2.55	50.05	64.79	15.41	99.31	2.04	Clostridiales	Firmicutes	Fournierella massiliensis
Sample_B17_1.bin_21	B171.M021	37	2.21	74.56	38.13	15.38	86.17	0.58	Lachnospiraceae	Firmicutes	Ruminococcus sp. B05
Sample_A07_3.bin_21	A073.M021	348	2.58	11.16	27.42	15.17	84.48	0.00	Bacteria	Firmicutes	Romboutsia ilealis
Sample_A32_2.bin_21	A322.M021	95	3.00	45.04	46.08	15.13	97.04	2.01	Bacteroidales	Bacteroidetes	Porphyromonadaceae bacterium
Sample_A31_2.bin_21	A312.M021	41	2.90	91.31	28.21	15.08	98.38	0.00	Clostridiales	Firmicutes	Clostridium perfringens
Sample_B16_1.bin_21	B161.M021	99	4.25	60.63	42.38	14.95	95.69	0.32	Bacteria	Bacteroidetes	Butyricimonas virosa
Sample_A05_3.bin_21	A053.M021	53	1.82	68.88	46.76	14.91	95.69	0.00	Bacteria	Proteobacteria	Azospirillum sp.
Sample_A07_2.bin_22	A072.M022	88	3.60	66.35	44.28	14.89	88.50	1.42	Bacteria	Firmicutes	Clostridium innocuum
Sample_B22_3.bin_22	B223.M022	72	1.98	40.16	57.28	14.68	87.46	0.00	Clostridiales	Firmicutes	Clostridiales bacterium
Sample_A27_1.bin_22	A271.M022	54	1.76	59.01	41.18	14.57	93.61	0.00	Clostridiales	Firmicutes	Mogibacterium diversum
Sample_A04_3.bin_22	A043.M022	84	2.88	54.80	35.77	14.54	99.32	0.67	Clostridiales	Firmicutes	Eubacterium sp.
Sample_A31_2.bin_22	A312.M022	221	3.44	28.32	64.42	14.54	100.00	0.81	Actinobacteria	Actinobacteria	Eggerthella lenta

Sample_B17_3.bin_22	B173.M022	34	2.28	96.71	45.40	14.49	96.61	0.74	Lachnospiraceae	Firmicutes	Roseburia sp. AM16-25
Sample_A04_1.bin_22	A041.M022	58	2.89	70.25	49.71	14.44	98.53	0.24	Lachnospiraceae	Firmicutes	Roseburia hominis
Sample_A29_1.bin_22	A291.M022	31	2.56	133.88	60.00	14.41	95.92	0.00	Bacteroidetes	Bacteroidetes	Alistipes communis
Sample_A31_1.bin_23	A311.M023	235	2.49	15.28	50.03	14.37	86.39	4.84	Clostridiales	Firmicutes	uncultured Clostridium sp.
Sample_A22_2.bin_23	A222.M023	146	2.56	39.19	48.65	14.30	100.00	0.11	Proteobacteria	Bacteroidetes	Proteobacteria bacterium CAG:139
Sample_A27_2.bin_23	A272.M023	70	1.27	28.40	24.96	14.25	91.57	1.69	Bacteria	Firmicutes	Clostridium sp. CAG:433
Sample_A33_2.bin_23	A332.M023	30	2.09	116.86	44.20	14.25	92.61	0.00	Clostridiales	Firmicutes	Frisingicoccus caecimuris
Sample_A27_1.bin_23	A271.M023	50	3.43	89.36	42.21	14.21	81.59	0.56	Bacteroidales	Bacteroidetes	Bacteroides sp. CAG:462
Sample_A03_2.bin_23	A032.M023	121	1.97	25.81	57.13	14.19	95.62	0.00	Proteobacteria	Proteobacteria	Sutterella sp. CAG:397
Sample_B15_1.bin_23	B151.M023	76	2.05	33.83	49.28	14.00	86.20	1.34	Lachnospiraceae	Firmicutes	uncultured Clostridium sp.
Sample_A24_2.bin_23	A242.M023	49	2.15	68.91	49.67	13.99	93.95	0.00	Clostridiales	Firmicutes	Ruminococcus sp. OM06-36AC
Sample_A27_2.bin_24	A272.M024	52	2.32	58.69	62.01	13.67	95.30	2.68	Clostridiales	Firmicutes	Lawsonibacter asaccharolyticus
Sample_A27_1.bin_24	A271.M024	53	2.28	62.74	57.62	13.62	91.05	0.67	Clostridiales	Firmicutes	Firmicutes bacterium CAG:137
Sample_B17_1.bin_24	B171.M024	48	2.58	90.54	63.44	13.38	99.19	0.00	Actinobacteria	Actinobacteria	Asaccharobacter celatus
Sample_B15_2.bin_24	B152.M024	62	2.33	55.09	46.92	13.26	93.04	3.29	Selenomonadales	Firmicutes	Phascolarctobacterium faecium

Sample_B24_3.bin_24	B243.M024	109	5.43	67.98	56.33	13.20	94.70	0.55	Enterobacteriaceae	Proteobacteria	Klebsiella oxytoca
Sample_A18_2.bin_24	A182.M024	38	1.70	58.40	48.64	12.92	89.14	1.01	Clostridiales	Firmicutes	Eubacterium sp. CAG:841
Sample_A32_2.bin_24	A322.M024	226	2.51	18.05	34.48	12.85	85.02	0.60	Bacilli	Firmicutes	Turicibacter sanguinis
Sample_B02_2.bin_24	B022.M024	57	2.65	80.46	54.98	12.71	98.38	0.00	Clostridia	Firmicutes	Clostridia bacterium
Sample_B16_1.bin_25	B161.M025	64	2.09	69.05	49.83	12.71	94.35	0.00	Clostridia	Firmicutes	Clostridiales bacterium
Sample_A05_2.bin_25	A052.M025	125	2.41	37.88	62.41	12.55	98.13	0.62	Proteobacteria	Proteobacteria	Sutterella wadsworthensis
Sample_A32_1.bin_25	A321.M025	67	3.39	65.46	45.38	12.51	87.50	0.00	Bacteroidales	Bacteroidetes	Parabacteroides johnsonii
Sample_B21_3.bin_25	B213.M025	52	2.18	68.37	44.60	12.38	97.09	0.00	Clostridiales	Firmicutes	Ruminococcus sp. CAG:488
Sample_B14_1.bin_25	B141.M025	75	2.58	46.01	44.48	12.36	87.60	1.55	Lachnospiraceae	Firmicutes	Lachnospiraceae bacterium
Sample_B12_2.bin_25	B122.M025	64	1.64	42.07	52.67	12.36	87.84	0.34	Clostridiales	Firmicutes	Firmicutes bacterium CAG:555
Sample_A23_2.bin_25	A232.M025	57	1.84	46.17	49.49	12.25	95.96	0.03	Clostridia	Firmicutes	Clostridiales bacterium
Sample_A04_3.bin_26	A043.M026	41	2.22	58.73	62.16	12.21	91.27	0.00	Clostridiales	Firmicutes	uncultured Oscillibacter sp.
Sample_A19_1.bin_26	A191.M026	255	3.11	16.98	56.04	11.95	97.96	0.00	Bacteria	Synergistetes	Cloacibacillus porcorum
Sample_A03_2.bin_26	A032.M026	103	2.12	34.71	62.95	11.91	99.02	2.37	Bifidobacteriaceae	Actinobacteria	Bifidobacterium bifidum
Sample_B15_1.bin_26	B151.M026	255	2.10	11.23	58.88	11.90	86.05	3.44	Clostridiales	Firmicutes	Faecalibacterium prausnitzii

Sample_B17_3.bin_26	B173.M026	57	1.65	49.75	43.13	11.86	91.72	0.00	Clostridiales	Firmicutes	Ruminococcaceae bacterium
Sample_A29_2.bin_26	A292.M026	87	1.84	34.32	52.68	11.85	97.95	0.00	Lactobacillales	Firmicutes	Lactobacillus fermentum
Sample_B21_2.bin_26	B212.M026	79	1.64	34.59	40.21	11.82	80.60	0.58	Streptococcus	Firmicutes	Streptococcus salivarius
Sample_A31_2.bin_26	A312.M026	150	2.05	19.35	56.59	11.78	91.39	0.67	Clostridiales	Firmicutes	Ruminococcaceae bacterium
Sample_B16_3.bin_26	B163.M026	81	2.32	39.07	45.39	11.65	86.20	2.51	Lachnospiraceae	Firmicutes	uncultured Clostridium sp.
Sample_A04_1.bin_26	A041.M026	68	1.95	33.88	57.20	11.38	94.73	0.67	Clostridiales	Firmicutes	Clostridiales bacterium
Sample_A04_2.bin_26	A042.M026	129	2.72	31.65	53.83	11.37	97.03	1.01	Clostridiales	Firmicutes	Ruminococcaceae bacterium
Sample_A01_2.bin_26	A012.M026	164	2.05	22.23	58.93	11.35	90.82	2.06	Clostridiales	Firmicutes	Butyricicoccus sp. OM04-18BH
Sample_A29_1.bin_27	A291.M027	78	1.51	31.41	46.70	11.25	93.43	0.00	Firmicutes	Firmicutes	Veillonellaceae bacterium
Sample_B16_1.bin_27	B161.M027	79	1.94	38.66	37.26	11.23	95.46	0.00	Clostridiales	Firmicutes	Ruminococcus sp. CAG:624
Sample_A23_2.bin_27	A232.M027	77	1.84	50.64	60.12	11.23	84.22	0.67	Clostridiales	Firmicutes	Evtepia gabavorous
Sample_A33_1.bin_27	A331.M027	187	1.79	12.74	53.62	11.18	96.15	3.29	Clostridia	Firmicutes	Clostridia bacterium
Sample_B17_1.bin_27	B171.M027	136	3.34	31.34	56.29	11.05	80.44	1.58	Enterobacteriaceae	Proteobacteria	Leclercia adecarboxylata
Sample_A28_3.bin_27	A283.M027	85	2.20	38.08	62.55	10.99	97.98	0.81	Actinobacteria	Actinobacteria	Olsenella sp. AF21-51
Sample_A27_1.bin_27	A271.M027	214	2.13	14.27	59.92	10.90	95.34	1.61	Actinobacteria	Actinobacteria	Collinsella aerofaciens

Sample_A03_2.bin_27	A032.M027	173	3.32	27.73	42.89	10.85	89.76	0.00	Bacteria	Bacteroidetes	Butyricimonas faecalis
Sample_A07_2.bin_27	A072.M027	119	1.74	29.36	58.14	10.84	90.21	0.00	Clostridiales	Firmicutes	Oscillibacter sp. 57_20
Sample_A31_3.bin_27	A313.M027	361	2.16	7.10	51.22	10.84	89.82	1.73	Clostridiales	Firmicutes	Clostridium leptum
Sample_B21_3.bin_27	B213.M027	99	2.36	45.87	58.73	10.81	99.20	0.98	Bifidobacteriaceae	Actinobacteria	Bifidobacterium sp. MSTE12
Sample_B15_1.bin_27	B151.M027	209	2.27	17.00	55.26	10.81	88.91	0.34	Clostridiales	Firmicutes	Clostridiales bacterium
Sample_A12_3.bin_27	A123.M027	164	2.94	23.93	46.27	10.80	90.31	2.31	Prevotella	Bacteroidetes	Prevotella sp. CAG:732
Sample_A27_2.bin_27	A272.M027	58	2.01	51.54	44.05	10.66	86.39	0.00	Clostridiales	Firmicutes	Clostridium sp. CAG:167
Sample_A22_2.bin_27	A222.M027	100	2.42	33.21	55.81	10.63	91.61	0.67	Clostridiales	Firmicutes	Clostridiales bacterium
Sample_A27_3.bin_28	A273.M028	458	2.81	8.03	43.55	10.56	88.19	2.48	Lactobacillales	Firmicutes	Enterococcus casseliflavus
Sample_B16_2.bin_28	B162.M028	68	2.10	43.67	46.39	10.55	82.24	0.88	Lachnospiraceae	Firmicutes	Sellimonas intestinalis
Sample_A33_2.bin_28	A332.M028	200	2.43	18.11	50.22	10.30	85.44	1.29	Clostridiales	Firmicutes	Clostridium sp. AF27-2AA
Sample_B21_3.bin_28	B213.M028	142	2.06	19.40	56.71	10.23	86.62	0.67	Clostridiales	Firmicutes	Clostridiales bacterium
Sample_A03_2.bin_28	A032.M028	571	4.51	10.95	52.21	10.16	94.79	3.73	Enterobacteriaceae	Proteobacteria	uncultured Citrobacter sp.
Sample_B11_2.bin_28	B112.M028	180	2.19	20.84	51.81	10.14	94.12	2.81	Clostridiales	Firmicutes	Butyricicoccus porcorum
Sample_A27_2.bin_29	A272.M029	423	4.71	14.79	51.03	10.08	91.94	1.38	Clostridiales	Firmicutes	Enterocloster aldensis

Sample_B17_1.bin_29	B171.M029	572	3.71	8.23	60.05	9.90	82.54	3.09	Actinobacteria	Actinobacteria	Eggerthella lenta
Sample_A09_2.bin_29	A092.M029	244	2.29	14.07	51.58	9.69	88.09	4.11	Clostridiales	Firmicutes	uncultured Clostridium sp.
Sample_B15_1.bin_30	B151.M030	368	2.83	10.29	46.02	9.69	84.57	3.76	Lachnospiraceae	Firmicutes	Lachnospiraceae bacterium
Sample_B16_1.bin_30	B161.M030	142	1.65	17.03	60.66	9.61	80.66	0.34	Clostridiales	Firmicutes	Evtepia gabavorous
Sample_A27_2.bin_30	A272.M030	182	2.32	19.81	44.74	9.54	96.64	0.00	Clostridiales	Firmicutes	Ruminococcaceae bacterium
Sample_A19_2.bin_30	A192.M030	338	1.79	6.60	57.11	9.53	82.36	2.07	Clostridiales	Firmicutes	Clostridium sp. CAG:242
Sample_B02_2.bin_30	B022.M030	168	1.90	20.26	57.63	9.53	86.18	2.37	Clostridiales	Firmicutes	Oscillibacter sp.
Sample_A03_2.bin_31	A032.M031	97	1.95	27.87	43.07	9.52	85.11	0.00	Clostridiales	Firmicutes	Clostridiales bacterium
Sample_B24_1.bin_31	B241.M031	177	1.70	12.30	49.23	9.50	96.23	0.00	Bacteria	Proteobacteria	Azospirillum sp. CAG:239
Sample_A32_1.bin_31	A321.M031	278	2.02	9.02	64.65	9.35	86.45	2.01	Clostridiales	Firmicutes	Oscillibacter sp. PC13
Sample_A23_3.bin_31	A233.M031	284	2.06	8.52	56.25	9.26	87.70	0.89	Clostridiales	Firmicutes	Ruminococcaceae bacterium AM28-23LB
Sample_A12_3.bin_32	A123.M032	173	1.93	14.37	58.66	9.02	85.79	1.74	Clostridiales	Firmicutes	Ruminococcaceae bacterium
Sample_B24_1.bin_32	B241.M032	332	2.15	8.71	63.58	9.02	90.47	0.75	Proteobacteria	Proteobacteria	Sutterella megalosphaeroides
Sample_A19_1.bin_33	A191.M033	494	3.97	11.89	65.19	8.99	93.06	3.51	Actinobacteria	Actinobacteria	Eggerthella sinensis
Sample_A31_2.bin_33	A312.M033	303	2.23	9.20	63.03	8.80	88.44	2.51	Clostridiales	Firmicutes	Clostridiales bacterium

Sample_A27_1.bin_34	A271.M034	353	2.14	7.51	50.60	8.61	83.73	0.71	Clostridiales	Firmicutes	Firmicutes bacterium CAG:238
Sample_A27_2.bin_35	A272.M035	363	2.25	7.76	52.37	8.48	80.62	3.76	Lachnospiraceae	Firmicutes	Lachnoclostridium sp. An118
Sample_A27_1.bin_37	A271.M037	243	1.74	8.96	33.27	8.00	86.72	0.06	Clostridiales	Firmicutes	Ruminococcus sp.
Sample_A03_1.bin_38	A031.M038	260	1.70	8.14	53.69	7.92	80.80	1.01	Clostridiales	Firmicutes	Ruminococcaceae bacterium
Sample_A31_2.bin_38	A312.M038	234	1.56	8.10	51.56	7.89	81.77	1.34	Clostridiales	Firmicutes	Clostridiales bacterium
Sample_A27_1.bin_49	A271.M049	216	1.48	8.68	57.83	7.87	80.11	0.00	Clostridiales	Firmicutes	Clostridia bacterium
Sample_A27_1.bin_56	A271.M056	325	1.59	5.48	39.63	7.23	82.53	1.78	Pasteurellaceae	Proteobacteria	Haemophilus parainfluenzae
