# Supplementary material for: Bifidobacterium lactis Probio-M8 Adjuvant Treatment Confers Added Benefits to Patients with Coronary Artery Disease via Target Modulation of the Gut-Heart/-Brain Axes
Source: mSystems. 2022 Mar 28;7(2):e00100-22. doi: 10.1128/msystems.00100-22 (PMC9040731; doi:10.1128/msystems.00100-22)
Supplement: TABLE S5 [file msystems.00100-22-st005.pdf]

Table S5. Significant differential species-level genome bins (SGBs) between probiotic and placebo groups at different time points
Remarks: pro, probiotic group; pla, placebo group

Probiotic group												
**Differential SGBs**		**statistics (Centered Log-ration transformed abun**						**Corrected P-value, T-test**				
SGBs ID	Taxonomy	Mean_pro_0d	Mean_pro_90d	Mean_pro_180d	SD_pro_0d	SD_pro_90d	SD_pro_180d	pro_0d vs pla_0d	pro_0d vs pro_90d	pro_0d vs pro_180d	pro_90d vs pro_180d
A222.M001	Bifidobacterium animalis	-0.63	0.97	1.05	0.49	2.42	2.14	0.240	0.042	0.022	0.838
A191.M001	Bacteroides sp. CAG:20	-0.31	0.62	-0.15	1.49	1.75	1.53	0.890	0.041	0.756	0.079
B201.M002	Eubacterium sp. CAG:115	0.20	-0.08	-0.19	0.94	0.39	0.00	0.352	0.351	0.039	0.131
B143.M007	Roseburia inulinivorans	0.61	-0.20	-0.14	1.35	1.63	1.36	0.243	0.066	0.039	0.871
A273.M017	Ruminococcaceae bacterium	-0.11	-0.04	0.39	0.30	0.58	1.13	0.800	0.333	0.040	0.292
A061.M008	Bifidobacterium bifidum	-0.12	-0.17	0.38	0.69	0.31	1.15	0.410	0.757	0.027	0.049
A073.M001	Bifidobacterium adolescentis	-0.19	-0.13	0.78	1.20	1.42	1.80	0.620	0.851	0.041	0.050
B163.M001	Alistipes sp. AF17-16	-0.15	0.43	-0.21	0.86	1.30	0.83	0.520	0.06	0.82	0.038

**Between the probiotic and placebo groups**														**Corrected P-value, Wilcoxon-test**		
SGBs ID	Taxonomy	Mean_pro_0d	Mean_pro_90d	Mean_pro_180d	Mean_pla_0d	Mean_pla_90d	Mean_pla_180d	SD_pro_0d	SD_pro_90d	SD_pro_180d	SD_pla_0d	SD_pla_90d	SD_pla_180d	pro_0d vs pla_0d	pro_90d vs pla_90d	pro_180d vs pla_180d
A061.M002	Eubacterium sp. CAG:76	0.34	0.67	0.47	-0.65	-0.73	-0.70	1.85	1.75	1.53	1.54	1.39	1.44	0.053	0.024	0.022

A222.M 001	Bifidobacterium animalis	-0.63	0.97	1.05	-0.78	-0.64	-0.54	0.49	2.42	2.14	0.00	0.56	0.96	0.240	0.010	0.039
A212.M 010	Prevotella copri	0.71	0.99	0.73	-1.21	-1.18	-1.03	3.26	3.34	3.50	2.83	2.84	3.07	0.056	0.013	0.083
A062.M 007	Ruminococcus sp. OM06-36AC	0.12	0.48	0.18	-0.32	-0.46	-0.32	1.28	1.56	1.47	0.67	0.28	0.61	0.260	0.031	0.260
B173.M 011	Fusicatenibacter saccharivorans	0.27	0.42	0.07	-0.41	-0.51	-0.14	1.51	1.46	1.47	1.59	1.57	1.64	0.100	0.034	0.780
A123.M 002	Coprococcus sp. OM04-5BH	-0.10	0.49	0.02	-0.09	-0.39	-0.10	1.01	1.78	1.37	1.23	0.00	1.18	0.840	0.048	0.790
A271.M 018	Clostridiales bacterium	0.15	0.17	0.08	-0.19	-0.19	-0.19	0.82	0.73	0.70	0.00	0.00	0.00	0.085	0.048	0.085
A282.M 004	Megamonas funiformis	0.07	0.63	0.23	-0.36	-0.60	-0.35	1.77	2.32	1.74	1.11	0.55	1.14	0.440	0.050	0.280
B061.M 015	Clostridiales bacterium	-0.07	0.28	0.51	-0.40	0.04	-0.66	1.15	1.32	1.41	1.23	1.61	0.73	0.250	0.440	0.049
A283.M 014	Lachnospiraceae bacterium	0.16	0.10	0.24	-0.25	-0.19	-0.26	0.81	0.83	0.91	0.35	0.35	0.23	0.057	0.430	0.027
A273.M 017	Ruminococcacea e bacterium	-0.11	-0.04	0.39	0.01	-0.17	-0.17	0.30	0.58	1.13	0.76	0.00	0.00	0.800	0.240	0.028
A073.M 001	Bifidobacterium adolescentis	-0.19	-0.13	0.78	-0.34	0.02	-0.33	1.20	1.42	1.80	1.05	1.70	1.06	0.620	0.860	0.033
A072.M 027	uncultured Clostridium sp.	-0.01	0.11	0.35	-0.26	-0.15	-0.22	1.05	1.07	0.83	0.98	0.99	0.86	0.520	0.390	0.046
A023.M 005	Roseburia sp. CAG:197	0.18	0.05	0.32	-0.26	-0.26	-0.26	0.98	0.88	1.29	0.00	0.00	0.00	0.051	0.085	0.048
A322.M 021	Subdoligranulum sp. 60_17	0.11	0.14	0.19	-0.25	-0.11	-0.25	0.90	0.93	1.02	0.00	0.31	0.00	0.085	0.660	0.048
A053.M 001	Eggerthella sp. CAG:298	-0.06	0.15	0.26	-0.18	-0.09	-0.22	0.58	0.91	1.09	0.25	0.34	0.16	0.850	0.910	0.050
A192.M 030	Butyricicoccus porcorum	0.06	-0.02	0.32	-0.18	-0.18	-0.15	0.68	0.60	0.94	0.00	0.00	0.14	0.140	0.140	0.050

A071.M 001	Parabacteroides distasonis	-0.27	-0.20	-0.54	0.37	0.46	0.60	1.24	0.82	1.11	1.17	0.96	1.01	0.088	0.045	0.054
B062.M 001	Bacteroides uniformis	-0.27	-0.15	-0.74	0.32	0.70	0.61	1.43	1.22	1.38	1.54	1.19	1.22	0.110	0.050	0.053
B182.M 012	Parabacteroides johnsonii	-0.19	-0.22	-0.16	0.23	0.35	0.23	0.44	0.32	0.60	1.14	1.22	1.15	0.143	0.050	0.158
B213.M 017	Flavonifractor plautii	-0.20	-0.30	-0.37	0.44	0.49	0.31	1.04	0.98	0.77	1.37	1.31	1.36	0.109	0.050	0.132
