# Supplementary material for: Bifidobacterium lactis Probio-M8 Adjuvant Treatment Confers Added Benefits to Patients with Coronary Artery Disease via Target Modulation of the Gut-Heart/-Brain Axes
Source: mSystems. 2022 Mar 28;7(2):e00100-22. doi: 10.1128/msystems.00100-22 (PMC9040731; doi:10.1128/msystems.00100-22)
Supplement: FIG S1 [file msystems.00100-22-sf001.pdf]

Bifidobacterium lactis Probio-M8 adjuvant treatment confers added benefits to patients with coronary artery disease via target modulation of the gut-heart/-brain axes

Baoqing Sun[d#], Teng Ma[a,b,c#], Yalin Li[a,b,c#], Ni Yang[a,b,c], Bohai Li[a,b,c], Xinfu Zhou[d], Shuai Guo[a,b,c], Shukun Zhang[e], Lai-Yu Kwok[a,b,c], Zhihong Sun[a,b,c], Heping Zhang[a,b,c*]

[a] Inner Mongolia Key Laboratory of Dairy Biotechnology and Engineering, Inner Mongolia Agricultural University, Hohhot, Inner Mongolia, China

[b] Key Laboratory of Dairy Products Processing, Ministry of Agriculture and Rural Affairs, Inner Mongolia Agricultural University, Hohhot, Inner Mongolia, China

[c] Key Laboratory of Dairy Biotechnology and Engineering, Ministry of Education, Inner Mongolia Agricultural University, Hohhot, Inner Mongolia, China

[d] Department of Cardiology, Weihai Municipal Hospital, Cheeloo College of Medicine, Shandong University, Weihai, Shandong, China

[e] Department of Pathology, Weihai Municipal Hospital, Cheeloo College of Medicine, Shandong University, Weihai, Shandong, China

Figure S1. Patient screening process and effects of probiotic intervention on host gut microbiota and clinical indicators. (a) Flow diagram of the patient screening process for their suitability of participating in the clinical trial and sample collection. (b) Significant effect size of specific species-level genome bins (SGBs) on the clinical indicators in the probiotic (Pro) and placebo (Pla) groups. (c) Multivariable association of SGBs and clinical features. Five-pointed stars illustrate the identified responsive SGBs in the probiotic and placebo groups.

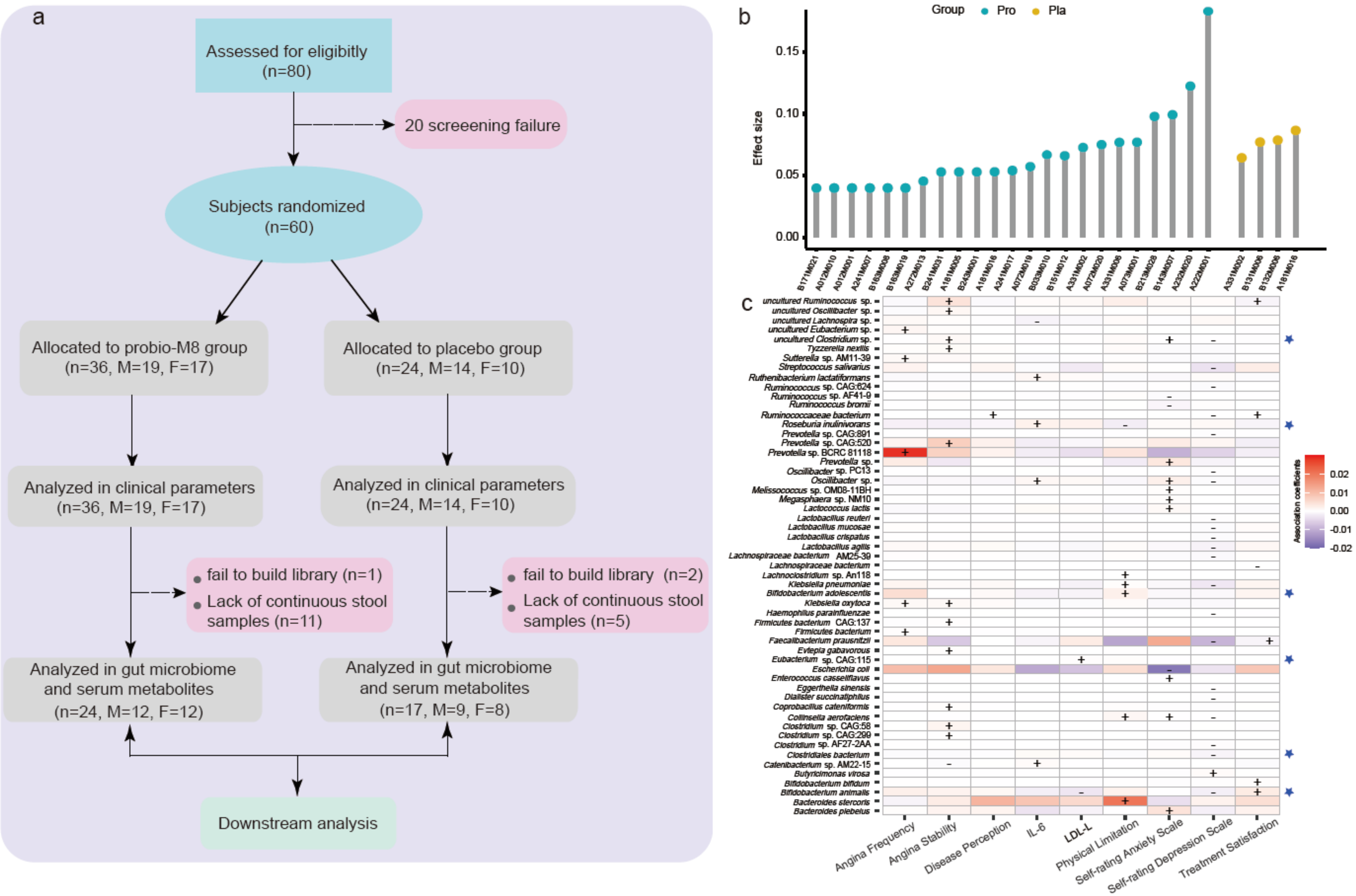

a
Assessed for eligibitly
(n=80)
20 screeening failure
Subjects randomized
(n=60)
Allocated to probio-M8 group
(n=36, M=19, F=17)
Allocated to placebo group
(n=24, M=14, F=10)
Analyzed in clinical parameters
(n=36, M=19, F=17)
Analyzed in clinical parameters
(n=24, M=14, F=10)
fail to build library (n=1)
Lack of continuous stool samples (n=11)
fail to build library (n=2)
Lack of continuous stool samples (n=5)
Analyzed in gut microbiome and serum metabolites
(n=24, M=12, F=12)
Analyzed in gut microbiome and serum metabolites
(n=17, M=9, F=8)
Downstream analysis
b
Group
Pro
Pla
Effect size
0.15
0.10
0.05
0.00
B171M021
A012M010
A012M001
A241M007
B163M008
B163M019
A272M013
B241M031
A181M005
B243M001
A181M016
A241M017
A072M019
B033M010
B151M012
A331M002
A072M020
A331M006
A073M001
B213M028
B143M007
A232M020
A222M001
A331M002
B131M006
B132M006
A181M016
c
uncultured Ruminococcus sp.
uncultured Oscillibacter sp.
uncultured Lachnospira sp.
uncultured Eubacterium sp.
uncultured Clostridium sp.
Tyzzerella nexilis
Sutterella sp. AM11-39
Streptococcus salivarius
Ruthenibacterium lactatiformans
Ruminococcus sp. CAG:624
Ruminococcus sp. AF41-9
Ruminococcus bromii
Ruminococcaceae bacterium
Roseburia inulinivorans
Prevotella sp. CAG:891
Prevotella sp. CAG:520
Prevotella sp. BCRC 81118
Prevotella sp.
Oscillibacter sp. PC13
Oscillibacter sp.
Melissococcus sp. OM08-11BH
Megasphaera sp. NM10
Lactococcus lactis
Lactobacillus reuteri
Lactobacillus mucosae
Lactobacillus crispatus
Lactobacillus agilis
Lachnospiraceae bacterium AM25-39
Lachnospiraceae bacterium
Lachnoclostridium sp. An118
Klebsiella pneumoniae
Bifidobacterium adolescentis
Klebsiella oxytoca
Haemophilus parainfluenzae
Firmicutes bacterium CAG:137
Firmicutes bacterium
Faecalibacterium prausnitzii
Evtepia gabavorous
Eubacterium sp. CAG:115
Escherichia coli
Enterococcus casseliflavus
Eggerthella sinensis
Dialister succinatiphilus
Coprobacillus cateniformis
Collinsella aerofaciens
Clostridium sp. CAG:58
Clostridium sp. CAG:299
Clostridium sp. AF27-2AA
Clostridiales bacterium
Catenibacterium sp. AM22-15
Butyricimonas virosa
Bifidobacterium bifidum
Bifidobacterium animalis
Bacteroides stercoris
Bacteroides plebeius
Angina Frequency
Angina Stability
Disease Perception
IL-6
LDL-L
Physical Limitation
Self-rating Anxiety Scale
Self-rating Depression Scale
Treatment Satisfaction
Association coefficients
0.02
0.01
0.00
-0.01
-0.02
