# Supplementary material for: Bifidobacterium lactis Probio-M8 Adjuvant Treatment Confers Added Benefits to Patients with Coronary Artery Disease via Target Modulation of the Gut-Heart/-Brain Axes
Source: mSystems. 2022 Mar 28;7(2):e00100-22. doi: 10.1128/msystems.00100-22 (PMC9040731; doi:10.1128/msystems.00100-22)
Supplement: FIG S2 [file msystems.00100-22-sf002.pdf]

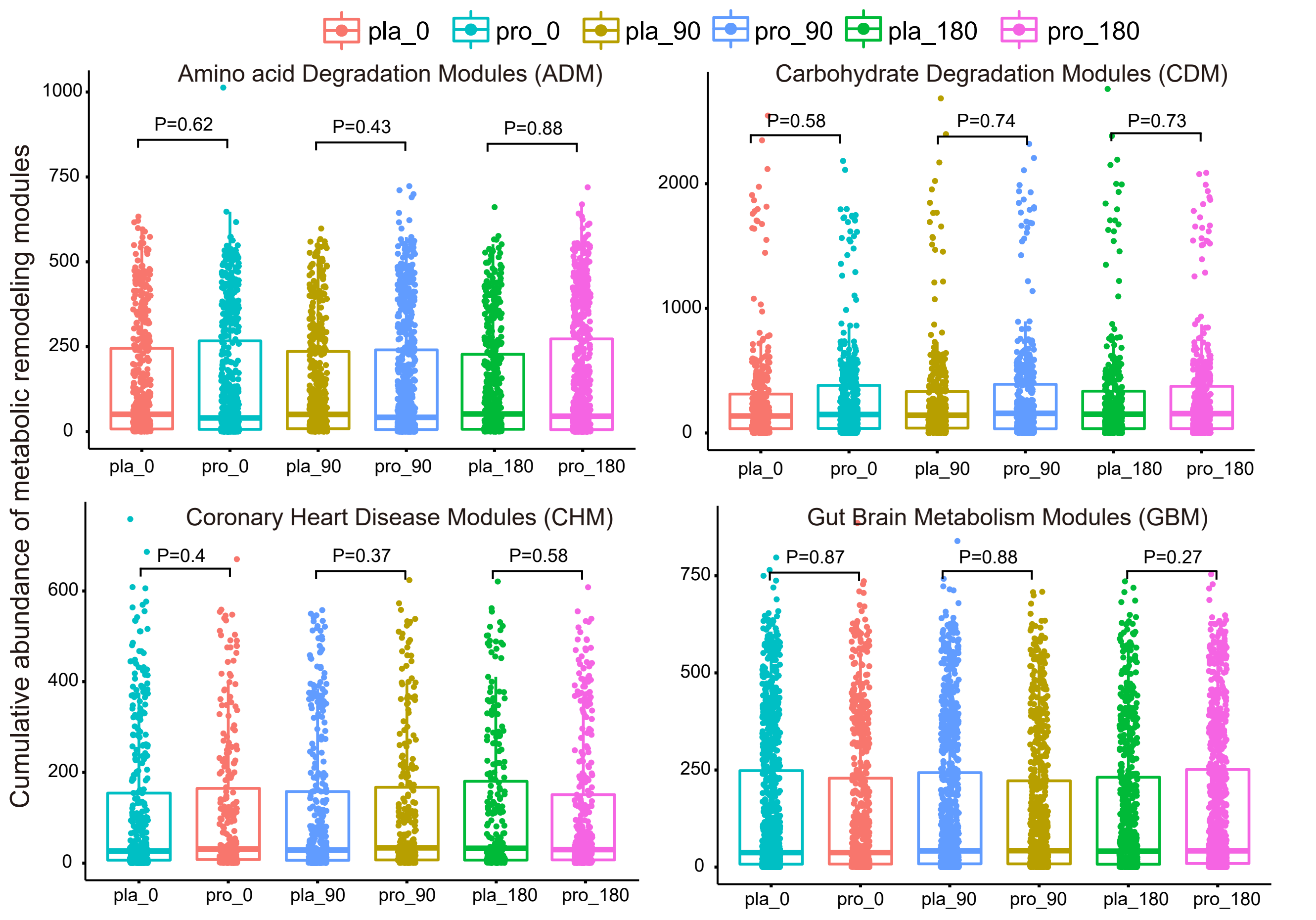
pla_0
pro_0
pla_90
pro_90
pla_180
pro_180
Cumulative abundance of metabolic remodeling modules
Amino acid Degradation Modules (ADM)
P=0.62
P=0.43
P=0.88
1000
750
500
250
0
pla_0
pro_0
pla_90
pro_90
pla_180
pro_180
Carbohydrate Degradation Modules (CDM)
P=0.58
P=0.74
P=0.73
2000
1000
0
pla_0
pro_0
pla_90
pro_90
pla_180
pro_180
Coronary Heart Disease Modules (CHM)
P=0.4
P=0.37
P=0.58
600
400
200
0
pla_0
pro_0
pla_90
pro_90
pla_180
pro_180
Gut Brain Metabolism Modules (GBM)
P=0.87
P=0.88
P=0.27
750
500
250
0
pla_0
pro_0
pla_90
pro_90
pla_180
pro_180
