# Supplementary material for: Bifidobacterium lactis Probio-M8 Adjuvant Treatment Confers Added Benefits to Patients with Coronary Artery Disease via Target Modulation of the Gut-Heart/-Brain Axes
Source: mSystems. 2022 Mar 28;7(2):e00100-22. doi: 10.1128/msystems.00100-22 (PMC9040731; doi:10.1128/msystems.00100-22)
Supplement: TABLE S6 [file msystems.00100-22-st006.pdf]

Table S6. Distribution of species-level genome bins (SGBs) and predicted metabolic modules across phyla

Module group	Module ID	Annotation	Total number of	Percentage (%)	Actinobacteria	Bacteroidetes	Melainabacteria	Cyanobacteria	Elusimicrobia	Euryarchaeota	Firmicutes	Fusobacteria	Lentisphaerae	Proteobacteria	Synergistetes	Tenericutes	Verrucomicrobia
	ADM001	phenylalanine degradation	21	4.77	0	0	1	0	0	0	1	0	0	18	0	0	1
	ADM010	isoleucine degradation	44	10.00	1	3	0	0	0	0	25	0	0	15	0	0	0
	ADM011	leucine degradation	9	2.05	1	5	0	0	0	0	3	0	0	0	0	0	0
	ADM012	methionine degradation I	294	66.74	17	41	2	1	1	1	193	1	1	31	2	1	3
	ADM013	methionine degradation II	38	8.64	3	0	0	0	0	0	28	2	0	4	1	0	0
	ADM014	proline degradation	35	7.95	1	12	0	0	0	0	3	0	0	15	1	0	3
	ADM015	valine degradation	25	5.68	1	0	0	0	0	0	9	0	0	15	0	0	0
	ADM017	cysteine biosynthesis/homocysteine degradation	22	5.00	0	0	0	0	0	0	13	0	0	9	0	0	0

**Amino acid degradation modules (ADM)**	ADM018	cysteine degradation I	415	94.32	24	47	2	1	1	1	302	2	0	29	2	1	3
	ADM019	cysteine degradation II	23	5.23	2	0	0	0	0	0	8	0	0	13	0	0	0
	ADM002	tyrosine degradation I	15	3.41	0	0	0	0	0	0	7	0	0	7	0	0	1
	ADM020	glutamine degradation I	77	17.50	2	31	0	0	0	0	26	2	0	13	0	0	3
	ADM021	glutamine degradation II	390	88.64	28	51	2	1	1	0	270	2	1	29	2	0	3
	ADM022	serine degradation	334	75.91	26	44	2	1	1	0	218	2	1	34	2	0	3
	ADM023	threonine degradation I	227	51.59	7	15	0	0	0	0	180	2	0	19	1	0	3
	ADM024	threonine degradation II	383	87.05	27	45	2	1	1	0	268	2	1	31	2	0	3
	ADM025	arginine degradation I	230	52.27	0	48	2	1	1	1	142	0	0	31	1	0	3

ADM026	arginine degradation II	14	3.18	0	0	0	0	0	0	0	0	0	14	0	0	0
ADM028	arginine degradation IV	31	7.05	0	12	0	0	0	0	11	0	0	7	0	0	1
ADM029	arginine degradation V	172	39.09	20	4	0	0	0	0	129	0	0	17	2	0	0
ADM003	tyrosine degradation II	11	2.50	0	0	0	0	0	0	3	1	0	7	0	0	0
ADM030	histidine degradation	65	14.77	0	24	0	0	0	0	25	1	0	13	2	0	0
ADM031	lysine degradation I	21	4.77	0	5	0	0	0	0	15	1	0	0	0	0	0
ADM032	lysine degradation II	31	7.05	0	0	1	1	0	0	13	0	0	15	1	0	0
ADM004	aspartate degradation I	318	72.27	28	50	1	0	1	1	211	2	1	18	2	0	3
ADM005	aspartate degradation II	129	29.32	5	29	0	0	0	0	61	2	1	31	0	0	0

ADM006	glutamate degradation III	21	4.77	0	0	0	0	0	0	13	2	0	6	0	0	0
ADM007	alanine degradation I	30	6.82	1	0	0	0	0	0	13	0	0	16	0	0	0
ADM008	alanine degradation II	79	17.95	6	31	0	0	0	0	38	1	0	1	2	0	0
ADM009	glycine degradation	114	25.83	2	42	2	1	1	0	43	0	1	18	1	0	3
CDM001	arabinoxylan degradation	227	51.59	6	32	0	0	0	0	175	1	1	10	0	0	2
CDM002	fructan degradation	191	43.41	13	32	0	0	0	0	129	1	1	15	0	0	0
CDM003	pectin degradation I	20	4.55	0	2	0	0	0	0	15	1	0	2	0	0	0
CDM004	pectine degradation II	40	9.09	0	11	0	0	0	0	23	1	0	5	0	0	0

**Carbohydrate degradation modules (CDM)**	CDM005	starch degradation	249	56.52	8	48	2	1	0	0	164	2	1	19	1	0	3
	CDM006	lactose degradation	290	65.91	19	49	0	0	0	0	200	1	1	17	0	0	3
	CDM007	lactose and galactose degradation	18	4.09	12	0	0	0	0	0	6	0	0	0	0	0	0
	CDM008	maltose degradation	26	5.91	0	5	0	0	0	0	21	0	0	0	0	0	0
	CDM009	melibiose degradation	234	53.18	7	38	0	0	0	0	172	1	1	12	0	0	3
	CDM010	sucrose degradation I	134	30.45	13	7	0	0	0	0	98	1	0	15	0	0	0
	CDM011	sucrose degradation II	55	12.50	6	0	0	0	0	0	32	0	0	14	0	0	3
	CDM012	trehalose degradation	67	15.23	1	4	0	0	0	0	46	1	1	14	0	0	0
	CDM013	allose degradation	19	4.32	1	0	0	0	0	0	12	0	0	6	0	0	0

	CDM014	arabinose degradation	126	28.64	5	20	0	0	0	0	86	0	1	14	0	0	0
	CDM015	fructose degradation	237	53.86	17	0	0	0	0	0	201	2	0	16	1	0	0
	CDM016	fucose degradation	58	13.14	0	0	0	0	0	0	41	2	1	11	0	0	3
	CDM017	galactose degradation	290	65.91	25	42	0	0	0	0	201	2	0	17	0	0	3
	CDM018	mannose degradation	227	51.59	19	47	0	0	0	0	134	2	1	21	0	0	3
	CDM019	rhamnose degradation	111	25.18	4	28	0	0	0	0	64	0	1	14	0	0	0
	CDM020	ribose degradation	55	12.50	2	0	0	0	0	0	37	2	0	14	0	0	0
	CDM021	xylose degradation	77	17.50	7	21	0	0	0	0	33	1	1	14	0	0	0

	CHM010	N-Acetyl-D-glucosamine 6-phosphate (GlcNAc-6-P) metabolism	438	99.55	29	50	2	1	1	1	310	2	1	35	2	1	3
	CHM002	choline degradation I	11	2.50	1	0	0	0	0	0	0	0	0	10	0	0	0
	CHM004	choline degradation III	22	5.00	0	1	0	0	0	0	13	0	1	6	1	0	0
	CHM006	L-carnitine degradation II	4	0.91	1	0	0	0	0	0	3	0	0	0	0	0	0
	CHM007	L-carnitine degradation III	6	1.36	0	0	0	0	0	0	0	0	0	6	0	0	0
	CHM011	creatine metabolism	75	17.05	7	9	0	0	0	0	47	1	0	9	2	0	0
	CHM012	mannitol metabolism	173	39.32	9	32	2	1	0	0	119	0	1	6	0	0	3
	CHM013	lysophosphatidylcholine metabolism	267	60.68	12	17	0	0	1	0	218	1	0	18	0	0	0
**Coronary heart disease**	CHM014	Acetate synthesis I	415	94.24	28	49	0	0	1	0	302	2	1	26	2	1	3

**disease modules (CHM)**	CHM015	Acetate synthesis II	3	0.68	0	0	0	0	0	0	3	0	0	0	0	0	0
	CHM016	Acetate synthesis III	30	6.82	0	9	1	0	0	0	17	0	0	0	1	0	2
	CHM018	Propionate synthesis I	8	1.82	0	0	0	0	0	0	0	0	0	8	0	0	0
	CHM019	Butyrate synthesis I	105	23.86	0	34	0	0	1	0	67	1	0	1	1	0	0
	CHM020	Butyrate synthesis II	29	6.59	0	5	0	0	0	0	22	2	0	0	0	0	0
	CHM021	Propionate synthesis II	48	10.91	1	0	0	0	0	0	40	2	0	4	1	0	0
	CHM022	Propionate synthesis III	51	11.59	1	33	0	0	0	0	12	0	0	0	2	0	3
	CHM023	Secondary bile acid biosynthesis I	8	1.82	1	3	0	0	0	0	1	2	0	0	1	0	0
	CHM024	Secondary bile acid biosynthesis II	26	5.91	2	0	0	0	0	0	24	0	0	0	0	0	0

	GBM001	Serotonin synthesis I	4	0.91	0	0	0	0	0	0	4	0	0	0	0	0	0
	GBM003	Melatonin synthesis	45	10.23	17	0	0	0	0	0	11	0	0	17	0	0	0
	GBM004	Kynurenine synthesis	1	0.23	1	0	0	0	0	0	0	0	0	0	0	0	0
	GBM005	Tryptophan synthesis	201	45.64	2	30	0	0	0	1	137	0	1	26	1	0	3
	GBM006	Glutamate synthesis I	383	87.05	29	50	0	1	1	1	273	1	1	21	2	0	3
	GBM007	Glutamate synthesis II	390	88.64	28	51	2	1	1	0	270	2	1	29	2	0	3
	GBM009	Histamine synthesis	7	1.59	3	2	0	0	0	0	1	1	0	0	0	0	0
	GBM010	Histamine degradation	10	2.27	1	0	0	0	0	0	0	0	0	9	0	0	0

	GBM011	Cortisol degradation	167	37.88	7	20	0	0	0	0	123	2	1	14	0	0	0
	GBM012	Dopamine synthesis	4	0.91	0	0	0	0	0	0	4	0	0	0	0	0	0
	GBM015	p-Cresol synthesis	224	50.91	5	32	1	1	1	0	144	2	1	32	2	0	3
	GBM016	p-Cresol degradation	6	1.36	1	0	0	0	0	0	0	0	0	5	0	0	0
	GBM017	IPA synthesis (Clostridium sporogenes pathway)	32	7.27	1	0	0	0	0	0	25	2	0	4	0	0	0
	GBM019	GABA degradation	61	13.86	6	0	0	0	0	0	39	0	0	15	1	0	0
	GBM020	GABA synthesis I	9	2.05	0	0	0	0	0	0	0	0	0	9	0	0	0
	GBM021	GABA synthesis II	13	2.95	0	0	0	0	0	0	0	0	0	13	0	0	0
	GBM022	GABA synthesis III	41	9.32	6	23	0	0	0	0	7	0	0	1	1	0	3

	GBM023	Dopamine degradation	16	3.64	1	1	0	0	0	0	5	0	0	9	0	0	0
	GBM024	DOPAC synthesis	9	2.05	1	1	0	0	0	0	2	0	0	5	0	0	0
	GBM026	Nitric oxide synthesis II (nitrite reductase)	3	0.68	0	0	0	0	0	0	0	0	0	3	0	0	0
	GBM027	Nitric oxide degradation I (NO dioxygenase)	33	7.50	0	1	0	0	0	0	7	1	0	24	0	0	0
	GBM028	Nitric oxide degradation II (NO reductase)	4	0.91	0	0	0	0	0	0	1	0	0	3	0	0	0
	GBM029	ClpB (ATP-dependent chaperone protein)	347	78.86	29	49	2	1	0	0	226	2	1	31	2	1	3
	GBM030	Polysaccharide A	15	3.41	0	6	0	0	0	0	9	0	0	0	0	0	0
	GBM031	17-beta-Estradiol degradation	320	72.73	10	44	1	0	0	0	230	2	1	28	0	1	3
**Gut-brain metabolism**	GBM032	Quinolinic acid synthesis	218	49.55	20	48	2	1	1	1	123	1	1	17	0	0	3

**modules (GBM)**	GBM033	Quinolinic acid degradation	415	94.24	29	50	2	1	1	1	292	2	1	31	2	0	3
	GBM034	Isovaleric acid synthesis I (KADH pathway)	3	0.68	0	1	0	0	0	0	2	0	0	0	0	0	0
	GBM035	Isovaleric acid synthesis II (KADC pathway)	126	28.56	22	10	1	0	0	0	80	0	1	11	1	0	0
	GBM036	S-Adenosylmethionine (SAM) synthesis	411	93.41	27	48	1	1	1	1	288	2	1	35	2	1	3
	GBM037	Inositol synthesis	19	4.32	5	14	0	0	0	0	0	0	0	0	0	0	0
	GBM038	Inositol degradation	95	21.59	18	9	0	0	0	0	56	0	1	11	0	0	0
	GBM039	g-Hydroxybutyric acid (GHB) degradation	50	11.36	1	4	1	0	0	0	34	0	0	9	1	0	0
	GBM040	Menaquinone synthesis (vitamin K2) I	71	16.14	7	34	0	0	0	0	9	0	0	18	0	0	3
	GBM041	vitamin K2 II	8	1.82	0	7	0	0	0	0	0	0	0	1	0	0	0

GBM042	PUFAs synthesis (AA, EPA, DHA)	275	62.50	4	24	1	0	1	0	206	1	1	35	2	0	0
GBM043	Acetate synthesis I	415	94.24	28	49	0	0	1	0	302	2	1	26	2	1	3
GBM044	Acetate synthesis II	3	0.68	0	0	0	0	0	0	3	0	0	0	0	0	0
GBM045	Acetate synthesis III	30	6.82	0	9	1	0	0	0	17	0	0	0	1	0	2
GBM047	Acetate degradation	116	26.36	5	29	0	0	0	1	65	0	1	15	0	0	0
GBM048	Propionate synthesis I	8	1.82	0	0	0	0	0	0	0	0	0	8	0	0	0
GBM049	Tryptophan degradation	41	9.32	0	15	0	0	0	0	18	0	0	5	0	0	3
GBM050	Glutamate degradation I	1	0.23	0	0	0	0	0	0	0	1	0	0	0	0	0
GBM051	Glutamate degradation II	21	4.77	0	0	0	0	0	0	13	2	0	6	0	0	0

	GBM052	Butyrate synthesis I	105	23.86	0	34	0	0	1	0	67	1	0	1	1	0	0
	GBM053	Butyrate synthesis II	29	6.59	0	5	0	0	0	0	22	2	0	0	0	0	0
	GBM054	Propionate synthesis II	48	10.91	1	0	0	0	0	0	40	2	0	4	1	0	0
	GBM055	Propionate synthesis III	51	11.59	1	33	0	0	0	0	12	0	0	0	2	0	3
	GBM056	Propionate degradation I	5	1.14	0	0	0	0	0	0	0	0	0	5	0	0	0
	GBM057	Corrinoid dependent enzymes	427	97.05	27	51	2	1	1	0	301	2	1	35	2	1	3
	GBM058	Secondary bile acid biosynthesis I	8	1.82	1	3	0	0	0	0	1	2	0	0	1	0	0
	GBM059	Secondary bile acid biosynthesis II	26	5.91	2	0	0	0	0	0	24	0	0	0	0	0	0
