# Supplementary material for: Bifidobacterium lactis Probio-M8 Adjuvant Treatment Confers Added Benefits to Patients with Coronary Artery Disease via Target Modulation of the Gut-Heart/-Brain Axes
Source: mSystems. 2022 Mar 28;7(2):e00100-22. doi: 10.1128/msystems.00100-22 (PMC9040731; doi:10.1128/msystems.00100-22)
Supplement: TABLE S7 [file msystems.00100-22-st007.pdf]

Table S7. Significant differential gut bioactive metabolites identified between probiotic and placebo groups

Probiotic group									
**Differential metabolite**	**Result statistics**						**Corrected P-value, T-test**		
	Mean_pr	Mean_pr	Mean_pr	SD_pro_	SD_pro_	SD_pro_	pro_0d	pro_0d	pro_90d
Pseudouridine	5.00E-05	4.66E-05	4.87E-05	5.51E-06	3.55E-06	4.28E-06	0.03	0.45	0.07
Fructose glucose galactose	1.39E-04	1.33E-04	1.35E-04	9.92E-06	8.28E-06	9.30E-06	0.04	0.24	0.60

placebo group									
**Differential metabolite**	**Result statistics**						**Corrected P-value, T-test**		
	Mean_pl	Mean_pl	Mean_pl	SD_pla_	SD_pla_	SD_pla_	pla_0d vs	pla_0d vs	pla_90d
Sebacate	1.20E-04	1.29E-04	1.02E-04	4.10E-05	4.51E-05	3.06E-05	0.61	0.15	0.05

Between the probiotic and placebo groups													**Corrected P-value,**		
**Differential metabolite**	Mean_pro 0d	Mean_pro 90d	Mean_pro 180d	Mean_pla 0d	Mean_pla 90d	Mean_pla 180d	SD_pro_0d	SD_pro_90d	SD_pro_180d	SD_pla_0d	SD_pla_90d	SD_pla_180d	pro_0d vs	pro_90d vs	pro_180d vs
X3 methylxanthine	3.11E-05	3.17E-05	3.13E-05	2.77E-05	2.73E-05	2.86E-05	7.55E-06	4.44E-06	5.25E-06	5.45E-06	6.59E-06	5.52E-06	0.13	0.02	0.19
C20:4 carnitine	1.20E-04	1.12E-04	1.11E-04	1.24E-04	1.27E-04	1.13E-04	3.32E-05	2.15E-05	2.86E-05	3.24E-05	3.05E-05	2.39E-05	0.47	0.04	0.63
Cytosine	2.95E-05	3.34E-05	3.17E-05	2.44E-05	2.26E-05	2.34E-05	1.18E-05	1.72E-05	1.22E-05	1.04E-05	9.95E-06	9.91E-06	0.20	0.05	0.04
Malonate	7.19E-05	6.95E-05	7.31E-05	6.58E-05	6.72E-05	6.59E-05	1.08E-05	8.47E-06	8.78E-06	5.05E-06	5.63E-06	5.28E-06	0.24	0.35	0.01
Palmitoyl glycerol	7.21E-05	7.22E-05	7.34E-05	7.00E-05	7.02E-05	7.07E-05	4.71E-06	4.23E-06	4.25E-06	3.34E-06	2.73E-06	2.75E-06	0.23	0.15	0.03
