# Supplementary material for: Bifidobacterium lactis Probio-M8 Adjuvant Treatment Confers Added Benefits to Patients with Coronary Artery Disease via Target Modulation of the Gut-Heart/-Brain Axes
Source: mSystems. 2022 Mar 28;7(2):e00100-22. doi: 10.1128/msystems.00100-22 (PMC9040731; doi:10.1128/msystems.00100-22)
Supplement: TABLE S8 [file msystems.00100-22-st008.pdf]

Table S8. Differential serum metabolites identified by LC-MS

Between the probiotic and placebo groups													Corrected P-value, Wilcoxon-test		
Serum metabolites (ng/ul)	Mean_pro_0d	Mean_pro_90d	Mean_pro_180d	Mean_pla_0d	Mean_pla_90d	Mean_pla_180d	SD_pro_0d	SD_pro_90d	SD_pro_180d	SD_pla_0d	SD_pla_90d	SD_pla_180d	pro_0d vs pla_0d	pro_90d vs pla_90d	pro_180d vs pla_180d
TMAO	44.05	48.98	34.08	64.32	68.34	79.73	28.60	32.11	14.76	44.59	41.70	56.49	1.00	0.52	0.03
L-Leucine	999.05	935.83	986.09	1081.68	1243.24	1386.87	438.12	452.31	484.21	481.69	576.90	525.54	1.000	0.060	0.019
L-Valine	1817.01	1684.75	1702.54	1660.63	1902.44	2079.87	467.36	422.03	318.55	797.54	724.14	492.36	1.000	1.000	0.017
L-Serine	33.58	32.84	42.63	30.12	34.89	29.47	15.75	15.44	14.07	14.58	22.31	16.22	1.000	1.000	0.008
L-Glycine	67.01	73.30	89.75	54.27	51.31	48.61	22.17	20.76	31.45	22.55	20.11	17.11	0.280	0.009	0.008
L-Cysteine	11.74	11.68	11.53	12.83	13.47	13.21	1.30	1.69	1.63	1.66	2.21	2.21	0.370	0.007	0.004
L-Arginine	132.68	118.96	113.22	206.88	212.05	218.17	83.20	90.49	131.93	145.17	148.86	161.29	0.650	0.037	0.004

Probiotic group									
	Result statistics						Corrected P-value, T-test		
Serum metabolites (ng/ul)	Mean_pro_0d	Mean_pro_90d	Mean_pro_180d	SD_pro_0d	SD_pro_90d	SD_pro_180d	pro_0d vs pro_90d	pro_0d vs pro_180d	pro_90d vs pro_180d
TMA	49.62	47.24	48.81	4.93	4.85	4.89	0.01	0.53	0.41
-Methionin	189.42	169.08	186.73	42.15	39.86	39.41	0.046	0.800	0.140
-Asparagir	44.13	38.99	45.56	13.67	13.06	12.67	0.100	0.520	0.030

Placebo group									
	Result statistics						Corrected P-value, T-test		

Serum metabolites (ng/ul)	Mean_pla_0d	Mean_pla_90d	Mean_pla_180d	SD_pla_0d	SD_pla_90d	SD_pla_180d	pla_0d vs pla_90d	pla_0d vs pla_180d	pla_90d vs pla_180d
-Methionin	174.69	211.22	187.56	95.37	93.15	103.49	0.040	0.480	0.310
-Kynurenir	15.36	20.22	25.89	12.02	11.51	19.09	0.048	0.042	0.240
