# Supplementary material for: Bifidobacterium lactis Probio-M8 Adjuvant Treatment Confers Added Benefits to Patients with Coronary Artery Disease via Target Modulation of the Gut-Heart/-Brain Axes
Source: mSystems. 2022 Mar 28;7(2):e00100-22. doi: 10.1128/msystems.00100-22 (PMC9040731; doi:10.1128/msystems.00100-22)
Supplement: TABLE S1 [file msystems.00100-22-st001.pdf]

Table S1. Information of subjects and samples

Subject number	Subject Information								
	Sample_ID	Gender	Age	Group	Group_time (pro: probiotic; pla: placebo)	BMI	Atorvastatin daily dosage (mg/d)	Fecal sample	Serum sample
	Sample_A01_1				pro_0d			√	√
1	Sample_A01_2	F	74	Probiotics	pro_90d	28.6	20	√	√
	Sample_A01_3				pro_180d			√	√
	Sample_A02_1				pro_0d			√	√
2	Sample_A02_2	M	65	Probiotics	pro_90d	23.7	20	√	√
	Sample_A02_3				pro_180d			√	√
	Sample_A03_1				pro_0d			√	√
3	Sample_A03_2	M	63	Probiotics	pro_90d	23.1	20	√	√
	Sample_A03_3				pro_180d			√	√
	Sample_A04_1				pro_0d			√	√
4	Sample_A04_2	F	61	Probiotics	pro_90d	28.6	20	√	√
	Sample_A04_3				pro_180d			√	√
	Sample_A05_1				pro_0d			√	√
5	Sample_A05_2	F	66	Probiotics	pro_90d	26.2	20	√	√
	Sample_A05_3				pro_180d			√	√
	Sample_A06_1				pro_0d			√	√
6	Sample_A06_2	M	66	Probiotics	pro_90d	32.9	20	√	√
	Sample_A06_3				pro_180d			√	√
	Sample_A07_1				pro_0d			√	√
7	Sample_A07_2	M	74	Probiotics	pro_90d	24.3	20	√	√
	Sample_A07_3				pro_180d			√	√
	Sample_A08_1				pro_0d			－	－
8	Sample_A08_2	M	73	Probiotics	pro_90d	24.7	20	－	－

Demographic data of two groups			
	Probiotics group	Placebo group	P-value
Male	16	20	－
Female	10	14	－
Age	65.11±8.14	66.29±10.31	0.93
BMI	25.11±2.27	25.56±2.04	0.27

	Sample_A08_3				pro_180d			–	–
	Sample_A09_1				pro_0d			√	√
9	Sample_A09_2	M	66	Probiotics	pro_90d	22.5	20	√	√
	Sample_A09_3				pro_180d			√	√
	Sample_A10_1				pro_0d			–	–
10	Sample_A10_2	M	70	Probiotics	pro_90d	24.4	20	–	–
	Sample_A10_3				pro_180d			–	–
	Sample_A11_1				pro_0d			–	–
11	Sample_A11_2	M	68	Probiotics	pro_90d	24.5	20	–	–
	Sample_A11_3				pro_180d			–	–
	Sample_A12_1				pro_0d			√	√
12	Sample_A12_2	F	67	Probiotics	pro_90d	24.2	20	√	√
	Sample_A12_3				pro_180d			√	√
	Sample_A13_1				pro_0d			–	–
13	Sample_A13_2	M	69	Probiotics	pro_90d	25.4	20	–	–
	Sample_A13_3				pro_180d			–	–
	Sample_A14_1				pro_0d			–	–
14	Sample_A14_2	M	67	Probiotics	pro_90d	26.1	20	–	–
	Sample_A14_3				pro_180d			–	–
	Sample_A15_1				pro_0d			–	–
15	Sample_A15_2	M	50	Probiotics	pro_90d	25.5	20	–	–
	Sample_A15_3				pro_180d			–	–
	Sample_A16_1				pro_0d			–	–
16	Sample_A16_2	F	58	Probiotics	pro_90d	23.4	20	–	–
	Sample_A16_3				pro_180d			–	–
	Sample_A17_1				pro_0d			√	√
17	Sample_A17_2	F	62	Probiotics	pro_90d	24.1	20	√	√
	Sample_A17_3				pro_180d			√	√
	Sample_A18_1				pro_0d			√	√
18	Sample_A18_2	M	66	Probiotics	pro_90d	25.3	20	√	√
	Sample_A18_3				pro_180d			√	√

	Sample_A19_1				pro_0d			√	√
19	Sample_A19_2	F	69	Probiotics	pro_90d	27.3	20	√	√
	Sample_A19_3				pro_180d			√	√
	Sample_A20_1				pro_0d			–	–
20	Sample_A20_2	F	66	Probiotics	pro_90d	23.3	20	–	–
	Sample_A20_3				pro_180d			–	–
	Sample_A21_1				pro_0d			√	√
21	Sample_A21_2	F	76	Probiotics	pro_90d	22.7	20	√	√
	Sample_A21_3				pro_180d			√	√
	Sample_A22_1				pro_0d			√	√
22	Sample_A22_2	M	71	Probiotics	pro_90d	27.1	20	√	√
	Sample_A22_3				pro_180d			√	√
	Sample_A23_1				pro_0d			√	√
23	Sample_A23_2	F	83	Probiotics	pro_90d	23.9	20	√	√
	Sample_A23_3				pro_180d			√	√
	Sample_A24_1				pro_0d			√	√
24	Sample_A24_2	M	60	Probiotics	pro_90d	22.4	20	√	√
	Sample_A24_3				pro_180d			√	√
	Sample_A25_1				pro_0d			–	–
25	Sample_A25_2	M	66	Probiotics	pro_90d	23.8	20	–	–
	Sample_A25_3				pro_180d			–	–
	Sample_A26_1				pro_0d			–	–
26	Sample_A26_2	F	75	Probiotics	pro_90d	23.2	20	–	–
	Sample_A26_3				pro_180d			–	–
	Sample_A27_1				pro_0d			√	√
27	Sample_A27_2	F	66	Probiotics	pro_90d	24.1	20	√	√
	Sample_A27_3				pro_180d			√	√
	Sample_A28_1				pro_0d			√	√
28	Sample_A28_2	M	65	Probiotics	pro_90d	24.7	20	√	√
	Sample_A28_3				pro_180d			√	√
	Sample_A29_1				pro_0d			√	√

29	Sample_A29_2	M	50	Probiotics	pro_90d	24.2	20	√	√
	Sample_A29_3				pro_180d			√	√
	Sample_A30_1				pro_0d			√	√
30	Sample_A30_2	F	50	Probiotics	pro_90d	27.3	20	√	√
	Sample_A30_3				pro_180d			√	√
	Sample_A31_1				pro_0d			√	√
31	Sample_A31_2	M	50	Probiotics	pro_90d	29.1	20	√	√
	Sample_A31_3				pro_180d			√	√
	Sample_A32_1				pro_0d			√	√
32	Sample_A32_2	M	52	Probiotics	pro_90d	26.1	20	√	√
	Sample_A32_3				pro_180d			√	√
	Sample_A33_1				pro_0d			√	√
33	Sample_A33_2	M	54	Probiotics	pro_90d	25.4	20	√	√
	Sample_A33_3				pro_180d			√	√
	Sample_A34_1				pro_0d			–	–
34	Sample_A34_2	F	62	Probiotics	pro_90d	21.1	20	–	–
	Sample_A34_3				pro_180d			–	–
	Sample_A35_1				pro_0d			–	–
35	Sample_A35_2	F	68	Probiotics	pro_90d	26.4	20	–	–
	Sample_A35_3				pro_180d			–	–
	Sample_A36_1				pro_0d			√	√
36	Sample_A36_2	F	76	Probiotics	pro_90d	24.3	20	√	√
	Sample_A36_3				pro_180d			√	√
	Sample_B01_1				pla_0d			–	–
37	Sample_B01_2	F	74	Placebo	pla_90d	25.4	20	–	–
	Sample_B01_3				pla_180d			–	–
	Sample_B02_1				pla_0d			√	√
38	Sample_B02_2	F	59	Placebo	pla_90d	28.1	20	√	√
	Sample_B02_3				pla_180d			√	√
	Sample_B03_1				pla_0d			√	√
39	Sample_B03_2	M	59	Placebo	pla_90d	25.8	20	√	√

	Sample_B03_3				pla_180d			√	√
	Sample_B04_1				pla_0d			–	–
40	Sample_B04_2	M	63	Placebo	pla_90d	24.9	20	–	–
	Sample_B04_3				pla_180d			–	–
	Sample_B05_1				pla_0d			√	√
41	Sample_B05_2	M	64	Placebo	pla_90d	28.2	20	√	√
	Sample_B05_3				pla_180d			√	√
	Sample_B06_1				pla_0d			√	√
42	Sample_B06_2	F	82	Placebo	pla_90d	28.5	20	√	√
	Sample_B06_3				pla_180d			√	√
	Sample_B07_1				pla_0d			–	–
43	Sample_B07_2	F	74	Placebo	pla_90d	27	20	–	–
	Sample_B07_3				pla_180d			–	–
	Sample_B08_1				pla_0d			–	–
44	Sample_B08_2	M	54	Placebo	pla_90d	27.8	20	–	–
	Sample_B08_3				pla_180d			–	–
	Sample_B09_1				pla_0d			–	–
45	Sample_B09_2	M	47	Placebo	pla_90d	21.1	20	–	–
	Sample_B09_3				pla_180d			–	–
	Sample_B10_1				pla_0d			–	–
46	Sample_B10_2	M	47	Placebo	pla_90d	26.3	20	–	–
	Sample_B10_3				pla_180d			–	–
	Sample_B11_1				pla_0d			√	√
47	Sample_B11_2	F	63	Placebo	pla_90d	23.9	20	√	√
	Sample_B11_3				pla_180d			√	√
	Sample_B12_1				pla_0d			√	√
48	Sample_B12_2	F	77	Placebo	pla_90d	24.6	20	√	√
	Sample_B12_3				pla_180d			√	√
	Sample_B13_1				pla_0d			√	√
49	Sample_B13_2	F	78	Placebo	pla_90d	27.1	20	√	√
	Sample_B13_3				pla_180d			√	√

	Sample_B14_1				pla_0d			√	√
50	Sample_B14_2	M	68	Placebo	pla_90d	27.3	20	√	√
	Sample_B14_3				pla_180d			√	√
	Sample_B15_1				pla_0d			√	√
51	Sample_B15_2	M	73	Placebo	pla_90d	26.1	20	√	√
	Sample_B15_3				pla_180d			√	√
	Sample_B16_1				pla_0d			√	√
52	Sample_B16_2	M	80	Placebo	pla_90d	25.3	20	√	√
	Sample_B16_3				pla_180d			√	√
	Sample_B17_1				pla_0d			√	√
53	Sample_B17_2	M	57	Placebo	pla_90d	28.6	20	√	√
	Sample_B17_3				pla_180d			√	√
	Sample_B18_1				pla_0d			√	√
54	Sample_B18_2	F	78	Placebo	pla_90d	25.1	20	√	√
	Sample_B18_3				pla_180d			√	√
	Sample_B19_1				pla_0d			–	–
55	Sample_B19_2	M	63	Placebo	pla_90d	23.2	20	–	–
	Sample_B19_3				pla_180d			–	–
	Sample_B20_1				pla_0d			√	√
56	Sample_B20_2	M	60	Placebo	pla_90d	23.5	20	√	√
	Sample_B20_3				pla_180d			√	√
	Sample_B21_1				pla_0d			√	√
57	Sample_B21_2	F	82	Placebo	pla_90d	24.2	20	√	√
	Sample_B21_3				pla_180d			√	√
	Sample_B22_1				pla_0d			√	√
58	Sample_B22_2	M	65	Placebo	pla_90d	22.6	20	√	√
	Sample_B22_3				pla_180d			√	√
	Sample_B23_1				pla_0d			√	√
59	Sample_B23_2	M	63	Placebo	pla_90d	23	20	√	√
	Sample_B23_3				pla_180d			√	√
	Sample_B24_1				pla_0d			√	√

60	Sample_B24_2	F	61	Placebo	pla_90d	25.8	20	√	√
	Sample_B24_3				pla_180d			√	√
