# Supplementary material for: Bifidobacterium lactis Probio-M8 Adjuvant Treatment Confers Added Benefits to Patients with Coronary Artery Disease via Target Modulation of the Gut-Heart/-Brain Axes
Source: mSystems. 2022 Mar 28;7(2):e00100-22. doi: 10.1128/msystems.00100-22 (PMC9040731; doi:10.1128/msystems.00100-22)
Supplement: TABLE S2 [file msystems.00100-22-st002.pdf]

Table S2. Information of multiple reaction monitoring-ion pair channel of detected metabolites of coronary artery diseases
Remarks: #Q1 resprent the parent ion of compound; *Q3 represent the daughter ion of the compound

Metabolites	Abbreviation of metabolites	Q1# (m/z)	Q3* (m/z)	De-clustering potential (DP; V)	Collision energy (CE; V)
Trimetlylamine oxide	TMAO	75.9	58.2	29.21	23.05
Trimethylamine	TMA	59.9	44.4	33.77	29.6
Lysine	Lys	147	84.1	28.3	21.61
Alanine	Ala	90.1	44.1	65.06	15.93
Isoleucine	Ile	131.9	69	20.79	22.81
Serine	Ser	105.8	60.1	33.98	13.37
Tryptophan	Trp	204.9	115	14.08	46.75
Phenylalanine	Pha	166	120.1	27.84	7.1
Leucine	Leu	132	86.1	13.88	13.27
Glycine	Gly	76.1	30	10	20.06
Glutamine	Gln	147	130	11.45	12.97
Threonine	Thr	120.1	74	16.03	13.29
Valine	Val	117.8	72	34.12	13.39
HIstidine	His	156.1	110	32	17.93
Proline	Pro	115.8	70.1	51.14	26.73
Amino oxalic acid	DL-2	161.9	97.9	16.92	20.18
Asparagine	Asn	133.1	74	24.03	19.21
Cystine	Cys-cys	240.9	152.1	40.11	18.34
Aspartic acid	Asp	134.1	73.9	16.26	17.97
Citrulline	Ccp	176.1	159.2	26.01	12.91
Arginine	Arg	175.2	69.9	43.85	25.02
Methionine	Met	150	103.8	15.74	13.9
Cysteine	Cys-2	122.2	76	17.17	17.04
Kynurenine	Kyn	209.1	94.1	28.77	18.21
Tyrosine	Tyr	182.1	165.1	13.81	12.65
